# Caudal fin shape imprinted during late zebrafish embryogenesis is re-patterned by the Sonic hedgehog pathway

**DOI:** 10.1371/journal.pbio.3003336

**Published:** 2025-08-25

**Authors:** Eric Surette, Joan Donahue, Crisvely Soto Martinez, Stephanie Robinson, Deirdre McKenna, Brendan Fitzgerald, Katherine Backus, Rolf O. Karlstrom, Nicolás Cumplido, Sarah K. McMenamin

**Affiliations:** 1 Boston College, Chestnut Hill, Massachusetts, United States of America; 2 University of Massachusetts, Amherst, Massachusetts, United States of America; University of Pennsylvania School of Medicine, UNITED STATES OF AMERICA

## Abstract

Appendage shape is formed during development–and re-established during regeneration–according to spatial and temporal cues that orchestrate local cell behaviors. The caudal fin is the primary appendage used for propulsion in most fishes, and the organ exhibits a range of distinct morphologies adapted for different swimming strategies. The external caudal fin of the zebrafish develops with a forked shape, with longer supportive bony rays at the periphery and shorter rays at the center of the organ. Here, we show that inducing a transient pulse of *sonic hedgehog a (shha)* overexpression during late embryonic development leads to excess growth of the central rays, causing the adult caudal fin to grow into a triangular, truncate shape. Our results identify a period–prior to endogenous *shha* expression and before differentiation of skeletogenic cells in these tissues–during which the imprinted fin shape can be re-patterned by hyper-physiological Shh stimulation. After this critical developmental period, overexpression of *shha* does not alter the shape of the adult caudal fin. Both global and local *shha* overexpression during the critical window of embryogenesis are sufficient to alter the fin shape, and a normal forked shape can be rescued by subsequent treatment with an antagonist of the canonical Shh pathway. The early pulse of *shha* expands *hox13* expression domains in the fin primordium, and leads to excessive proliferation in the central regions of the fin. After developing with a truncate shape, a truncate morphology was remembered and rebuilt during regeneration, suggesting that the shape imprinted during embryogenesis informs both developmental and regenerative morphogenesis. Ray-finned fishes have evolved a wide spectrum of caudal morphologies, and the current work offers insights into the developmental time periods and processes that inform growth and ultimate shape of the fin.

## Abstract 2

Caudal fins show great variation in shape between diverse fish species. This study shows that developmental cues in embryonic tissues involving the sonic hedgehog pathway imprint fin morphology, which can later shape the adult caudal fin in zebrafish, highlighting a critical window for modulating appendage morphology.

## Introduction

The development and regeneration of biological shapes requires precise deployment and temporal interpretation of spatial signals [1,2], and deviations in these processes can significantly alter ultimate organ phenotype and function [3]. The homocercal caudal fin varies in phenotype across ray-finned fishes, and is considered a major evolutionary innovation of teleosts [4–7]. The caudal fin shows an elegant external skeletal structure, complex enough to be developmentally informative, yet simple enough that essential aspects of form may be geometrically and mechanistically disentangled.

The external morphology of the caudal fin is formed by the relative lengths of the bony rays (lepidotrichia), and the difference in length between the outer dorsal and ventral (peripheral) rays and the central rays establishes the overall external shape. Caudal fin shape varies considerably across species with different swimming ecologies, corresponding to different hydrodynamic tradeoffs [8–10]. There are two major classes of fin shapes. Truncate fins exhibit central rays as long or longer than the peripheral rays, as in medaka, trout and many killifish; these morphologies provide a large surface area for rapid acceleration and maneuvering [9–11]. The other major category of fin includes forked shapes like those in tuna and many carp, in which the central rays are markedly shorter than the peripheral rays; these shapes likely maximize stability and efficient cruising [8,9,12,13].

Zebrafish have a caudal fin that exhibits a distinctly forked shape, and this organ is intensively studied as a model for skeletal growth, patterning and regeneration [14–18]. The zebrafish caudal fin is characterized by mirror-image symmetry of fin rays, reflected around the central hypural diastema, a cleft separating the central-most endoskeletal elements [4,7,17,19]. The external symmetry of the fin rays contrasts with the highly asymmetric internal supportive endoskeleton, where most rays are supported by modified ventral spines called hypurals [20,21]. During development, fin rays initially appear at what will become the center of the caudal fin, ventral to the notochord. Rays ossify in pairs around the hypural diastema, with increasingly peripheral rays appearing later [17,22]. The notochord flexes upward as the fin develops, re-orienting the organ from ventral to posterior, and establishing the externally symmetrical shape [4,17,19]. Zebrafish fins are highly regenerative appendages even through adulthood, regrowing to their original size and forked shape within weeks of amputation [18,23,24].

Decades of research have identified many of the developmental pathways that regulate morphogenesis, outgrowth and patterning of the caudal fin [25–29]. Posteriorly-expressed Hox genes imprint identities that govern fin ray length and number [7,30]. Early outgrowth of the caudal fin is initiated by pulses of cell proliferation at the distal end of the caudal fin fold mesenchyme [31,32]. As rays continue to grow, activity of ion channels and gap junctions regulate the speed and extent of skeletal growth by modulating tissue-level bioelectric-calcineurin signals [33–37]. During later outgrowth of the fin rays, thyroid hormone and relative activities of skeletogenic cells regulate proximodistal patterning of the rays and location of skeletal bifurcations [38,39]. While Sonic hedgehog (Shh) is not endogenously involved in the early stages of caudal fin formation, after the rays form, *shha* is expressed at the growing distal tips of outgrowing lepidotrichia [40–42], supporting ray bifurcation by trafficking pre-osteoblasts distally with migrating basal epidermis [42–44]. Disrupting calcineurin, thyroid hormone or Shh pathways during developmental or regenerative outgrowth can dramatically alter different aspects of fin morphology, but invariably, despite modifications to length or patterning of the rays, the forked shape of the fin remains consistent, with the relatively shortest rays at the center [7,33,38,44,45]. Thus, the pathways and developmental periods responsible for imprinting the forked shape of the caudal fin have remained unresolved.

The Shh pathway regulates growth and axis identity of vertebrate appendages, a role that predates the fin-to-limb transition. In tetrapod limb buds, Shh produced by the zone of polarizing activity (ZPA) is both necessary and sufficient to imprint posterior identity in the developing appendage [46–49]. Shh-producing ZPA-like regions are present in the posterior regions of pectoral and pelvic (paired) fins as well as dorsal and anal (median) fins of chondrichthyans [50,51] and bony fishes [52–54]. In contrast, no region resembling a ZPA has been identified in the developing caudal fin [40,55]. Although Shh regulates fin ray morphogenesis at later stages of development, the pathway does not normally contribute to the establishment or early patterning of the zebrafish caudal fin [40]. Nonetheless, here we demonstrate that hyperactivity of the Shh/Ptch pathway in the embryonic fin primordium can imprint a novel caudal fin shape, inducing overgrowth in central rays that shift the adult zebrafish caudal fin from a forked to a truncate morphology.

## Results

### A pulse of *shha* overexpression during embryonic development alters shape of the adult caudal fin

**Fig 1:**
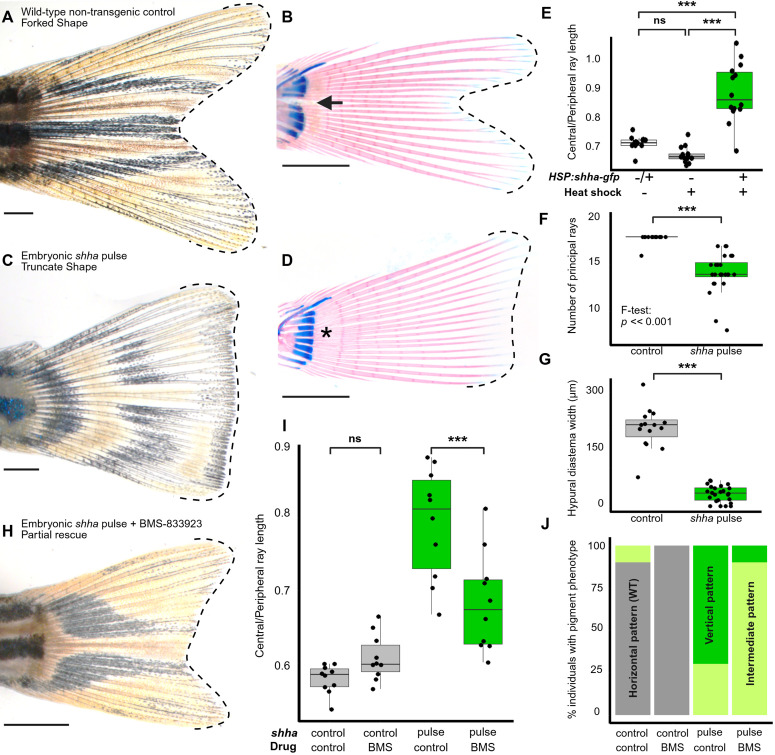
Premature pulse of *shha* during early fin fold development alters adult caudal fin shape. **(A–B)** Caudal fins of adult WT control and **(C–D)** transgenic zebrafish treated with pulse of *shha* overexpression at 2 dpf. (**B and D**) show cleared and stained caudal fins from juvenile zebrafish. Dashed outlines indicate the overall shape of the fins. Arrow indicates the location of the hypural diastema separating the dorsal from ventral lobes in **(B)**; asterisk indicates the absence of the diastema in **(D)**. **(E)** Both the *hsp70l:shha-eGFP* transgene and activation heat shock are to induce the truncate fin phenotype. Significance determined by ANOVA followed by Tukey’s post hoc test. **(F–G)** An embryonic *shha* pulse (**F**) increases the number and variance of principal fin rays and **(G)** causes a loss of the hypural diastema. Significance determined using Welch’s two-tailed *T*-tests. **(H–J)** Treatments with the Smoothened inhibitor BMS-833923 after *shha* pulse partially rescues both (**I**) forked fin shape and (**J**) horizontal stripes of pigmentation. Significance in (**I**) determined by ANOVA followed by Tukey’s post hoc test.Here and throughout, each datapoint on graphs **(E–G and I)** represents measurements from a single individual fish. Scale bars, 1 mm. The data underlying the graphs shown in the figure can be found in S1 Data and the summary statistics in S2 Data.

In the caudal fins of wild-type (WT) zebrafish, the shortest central rays are ~ 65% the length of the longest peripheral rays, creating the forked shape (Fig 1A–1B and 1E). We used a heat-shock-inducible *hsp70l:shha-EGFP* transgenic zebrafish line [56] to drive transient *shha* overexpression at 2 days post-fertilization (2 dpf; approximately 3 mm SL), well before skeletogenic lineages have differentiated in the caudal fin primordium [17,22]. We refer to this treatment of *shha* overexrpesssion during embryogenesis as "*shha* pulse;" clutch mates negative for the transgene but subjected to heat shock were used as controls. As adults, fish that had undergone embryonic *shha* pulse grew central caudal fin rays that were approximately 30% longer than control counterparts, reaching nearly the same length as the peripheral rays (often >85% the length of the peripheral rays). This excess central ray growth resulted in a truncate, triangular fin shape reminiscent of the caudal fins of medaka or killifish (Fig 1C–1E). The *shha* pulse caused a slight delay in relative fin growth during development (S1 Fig). However, by adult stages, the longest rays in truncate fins were as long as those in control fins (two-tailed *t* test: *p* = 0.3, and see S1 Fig); treatment with *shha* pulse changed fin shape without affecting adult fin length. Fin shape showed no correlation with sex (S2 Fig). Fish treated with *shha* pulse often developed fewer principal rays (Fig 1F), and showed a reduction or loss of the hypural diastema between hypurals 2 and 3 (Fig 1D and 1G). The dorsal, anal and paired fins showed no shifts in fin shape (S3 Fig); however, approximately 25% fish treated with *shha* pulse showed a reduced or absent anal fin (S4 Fig).

We asked if the effects of the *shha* pulse on the adult caudal fin shape were mediated via the canonical Shh signaling pathway. To test this, we inhibited the downstream Shh effector Smoothened with the antagonist drug BMS-833923 [43,44,57,58] at 24 and 48 hrs following the *shha* pulse. This inhibition partially rescued a forked shape (Fig 1H–1I), indicating that the *shha* pulse-induced truncate fin is dependent, at least in part, by hyperactivity of Shh/Smo signaling. In addition to the change in fin shape, treatment with *shha* pulse caused dramatic shifts in pigment pattern: truncate fins developed disorganized stripes often oriented in vertical arches or bars rather than the typical pattern of horizontal stripes (Fig 1A and 1C; also see S1B Fig); these pigment aberrations could be partially rescued by treatment with BMS-833923 following *shha* pulse (Fig 1H and 1J).

### Fin shape is imprinted in the embryonic fin during a critical period, which is sensitive to *shha* overexpression in a dose-dependent manner

**Fig 2:**
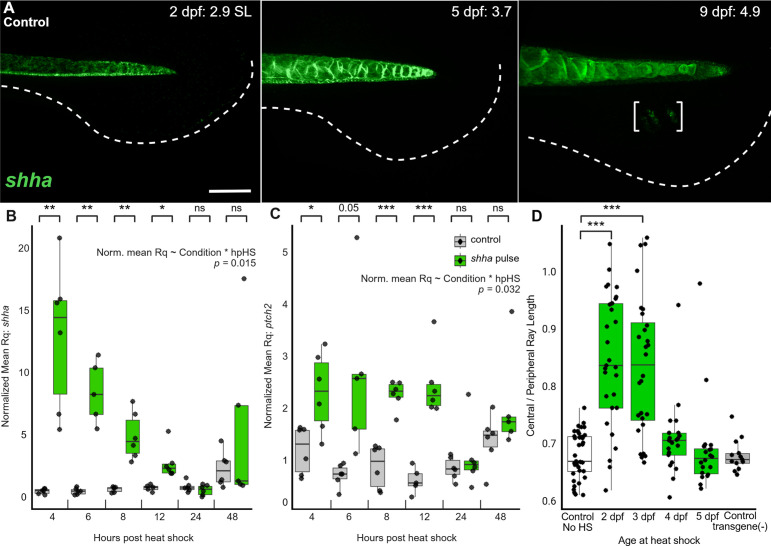
Transient overexpression of Shh pathway prior to native *shha* expression in the caudal fin primordium is sufficient to induce truncate fin shape. **(A)** Endogenous *shha* expression in developing WT caudal fins. At 9 dpf, *shha* expression is detectable in the fin tissue where the rays are beginning to develop (brackets). Dashed line indicates approximate ventral edge of fin folds. Staining in floor plate is genuine and some non-specific fluorescence is visible in the notochord vacuoles. Between 3 and 9 individuals were examined for each time point. Scale bar, 100 µm. **(B–C)** Duration of (**B**) *shha* and (**C**) *ptch2* overexpression, assessed 4–48 hrs following heat shock. Each datapoint represents the average Rq of three larvae pooled as a single biological replicate, normalized to a single replicate in the control group. Significance within timepoints determined using Welch’s two-tailed *T*-tests, and the correlation between trend and time following heat shock was determined by linear-mixed effects model. (**D**) *shha* pulse was induced at 2-5 dpf and only resulted in the development of a truncate fin shape only when heat shock is induced at 2 or 3 dpf. Heat shocks initiated at 1 dpf resulted in death and are not shown. Significance determined by ANOVA followed by Tukey’s post hoc tests. The data underlying the graphs shown in the figure can be found in S1 Data and the summary statistics in S2 Data.

In WT zebrafish at 2 dpf and 5 dpf, *shha* mRNA is present in the notochord and floor plate of the neural tube (Fig 2A). *shha* expression is first detectable in the caudal fin fold region as the first fin rays emerge (9 dpf, Fig 2A; also see [54]). We asked how long *shha* mRNA induced by the *shha* pulse perdured following the brief heat shock induction at 2 dpf. We found that at four hours following heat shock*,* transgenic animals showed 5–20 times more *shha* transcript than non-transgenic controls; *shha* mRNA decreased back to baseline levels by 24 hrs after heat shock (Fig 2B). The activation of the Shh pathway, as measured by *ptch2* transcript abundance [56,59], also returned to baseline 24 hrs after the *shha* pulse (Fig 2C). We conclude that the Shh pathway is only briefly hyper-activated by *shha* pulse, and returns to baseline levels by 3 dpf, many days before endogenous *shha* is normally expressed in the WT fin primordium.

To discern the critical period during which fin shape can be altered, we induced *shha* pulse at a range of different developmental time points. While *shha* pulse induced at 2 or 3 dpf was sufficient to induce a truncate phenotype, heat shock induction later at 4 or 5 dpf permitted the development of forked fins (Figs 2D and S5A). We asked whether this pattern could be explained by a failure of the transgenic promoter to function at later stages of development; however, we confirmed that later heat shocks remained effective at inducing GFP expression, causing overexpression of *shha* mRNA and inducing hyperactivation of the Shh pathway (S5B–S5D Fig). Together, these data suggest that development through 3 dpf (approximately 3.5 SL) constitutes a critical period during which hyper-physiological Shh pathway activity is sufficient to alter the fate of adult caudal fin shape.

**Fig 3:**
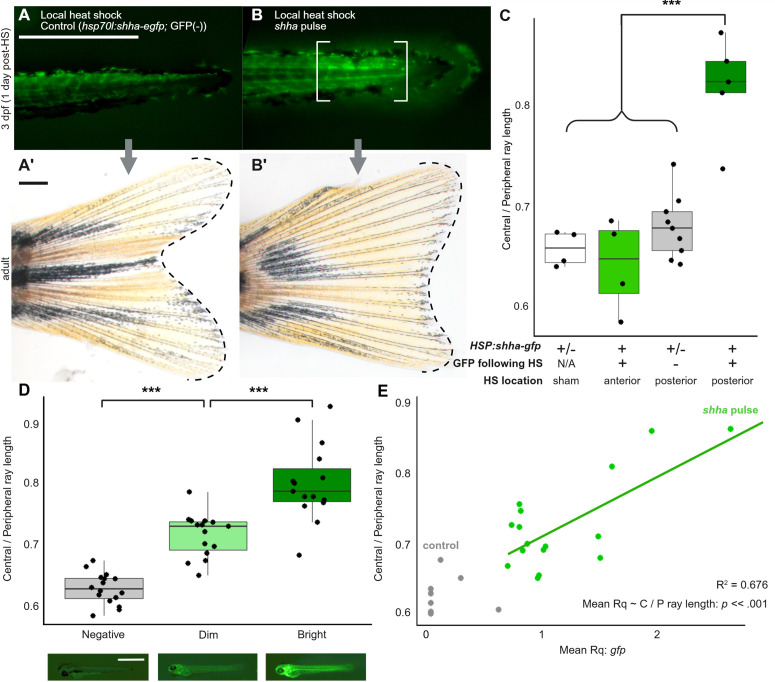
Embryonic *shha* pulse alters caudal fin development in a spatially-specific and dose-dependent manner. **(A–C)** Locally-induced *shha* pulse is sufficient to induce truncate phenotype. (**A–A’**) Embryo subjected to local posterior heat shock at 2 dpf did not show GFP fluorescence and grew into an adult with a forked fin. **(B–B’)** Local posterior heat shock induced GFP in transgenic embryo (brackets), which grew into an adult with a truncate fin. Scale bars, 500 µm. **(C)** Local heat shocks in transgenic embryos at posterior—but not anterior locations—can induce truncate fin shape. Significance determined by ANOVA followed by Tukey’s post hoc test. **(D)** Fish sorted by relative brightness of GFP expression 1 day after whole-body heat shock (4 dpf) show different caudal fin shapes as adults. Shown below the graph are representative images of individuals in each category. Significance determined by ANOVA followed by Tukey’s post hoc test. Scale bar, 1 mm. (**E**) *gfp* transgene abundance correlates with adult caudal fin shape. Significance between mean Rq and caudal fin shape is determined by linear-mixed effects model. The data underlying the graphs shown in the figure can be found in S1 Data and the summary statistics in S2 Data.

Given the local paracrine action of Shh [60,61] and that the majority of cells contributing to the caudal fin originate from the posterior end of the body axis [17,62], we predicted that *shha* overexpression restricted to the posterior region of the body would be sufficient to induce a truncate fin shape. To test this, we activated the *hsp70l:shha-gfp* transgene by local heat shock [63] at anteroposterior locations along the axis. Consistent with our prediction, local overexpression of *shha* specifically in the posterior end of the tail was sufficient to induce truncate morphologies, while anterior heat shock permitted development of a forked fin shape (Fig 3A–3C).

Transgenic embryos treated with *shha* pulse developed a range of truncate phenotypes as adults (see Figs 1E and 2D), and we speculated that the severity of the truncate phenotype was related to transgene copy number. Indeed, fluorophore brightness 1 day after heat shock induction as well as abundance of genomic *gfp* both predicted adult fin shape (Fig 3D–3E). We conclude that the shape information imprinted in the embryonic caudal fin is sensitive to *shha* overexpression in a dose-dependent manner.

### *shha* pulse expands *hox13* expression domains, disrupts ray development and prevents formation of a hypural diastema

**Fig 4:**
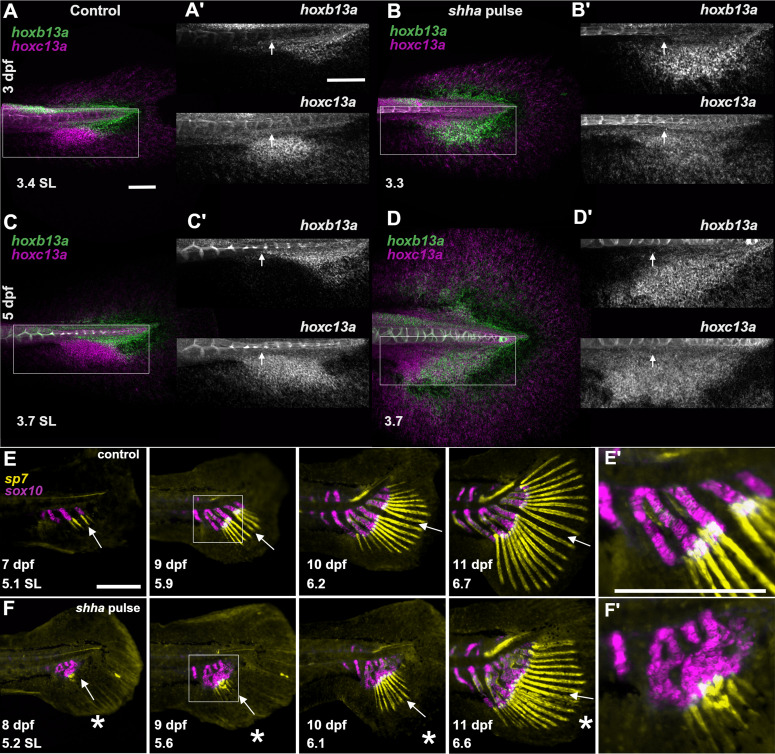
Early larval *hox13* expression and skeletogenesis are altered following *shha* pulse. **(A–D)** Domains of *hoxb13a* and *hoxc13a* in the caudal fin fold at 3 dpf (**A–B**) and 5 dpf (**C–D**) in non-transgenic controls (**A, C**) and transgenic individuals treated with *shha* pulse **(B, D)**. (**A’–D’**) shows higher magnification images of single *hox* targets within boxed areas. Small white arrow indicates the posterior end of the caudal artery. Standard lengths reported are corrected after fixation [22]. Between 3 and 9 individuals were examined for each condition and each time point. Scale bars, 100 µm. **(E–F)** Individuals imaged repeatedly in developing caudal region in (**E**) WT control and (**F**) larva treated with *shha* pulse. *sp7*-expressing osteoblasts shown in yellow; *sox10*-expressing chondrocytes shown in magenta. Arrow indicates the hypural diastema or where the diastema was expected (asterisk). Image series performed on a minimum of 6 larvae per condition. (**E’***)* and (**F’**) show higher magnification images of boxed areas at 9 dpf. Scale bars, 500 µM.

Zebrafish *hox13* genes regulate development of the posterior body axis and regional identity in the caudal fin elements [7,64]. *hoxa13b*, *hoxb13a and hoxc13a* occupy distinct, non-overlapping anteroposterior expression domains ventral to the notochord (Figs 4A, 4C, and S6; [7]); *hoxd13a* expression is mostly observed in the notochord (S6 Fig). Hox13 factors interact with the Shh pathway during development of both paired fins and tetrapod limbs [65–67], and we asked if *hox13* expression patterns were sensitive to *shha* overexpression. In larvae treated with *shha* pulse, we found that the expression domains of *hoxb13a* and *hoxc13a* were expanded across the fin fold, and the region of overlap between the genes was greatly enlarged (Figs 4B, 4D, and S6). Notably, *hoxb13a*, which is normally expressed posterior to the caudal artery (Fig 4A and 4C; [7]), showed expansion into progressively anterior regions (Fig 4B and 4D). Following *shha* pulse, *hoxd13a* also increased in expression at 3 dpf, while *hoxa13b* showed both expanded and more diffuse expression at 5 dpf (S6 Fig).

To test the effects of the *shha* pulse on later skeletogenesis, we tracked fin ray ossification and hypural chondrogenesis throughout larval development (Fig 4E–4F). In WT larvae, hypurals appear from anterior to posterior, while fin rays ossify sequentially in pairs around the hypural diastema from central to peripheral (Fig 4E and see [17,19]). In fish treated with *shha* pulse, the hypural complex was malformed and lacked a diastema from the earliest stages of chondrogenesis (Fig 4F). The appearance and growth of the fin rays was delayed and disordered, and no gap was maintained between the central rays (Fig 4F).

### Adult caudal fin shape is sculpted by regional differences in ray growth and cell proliferation

**Fig 5:**
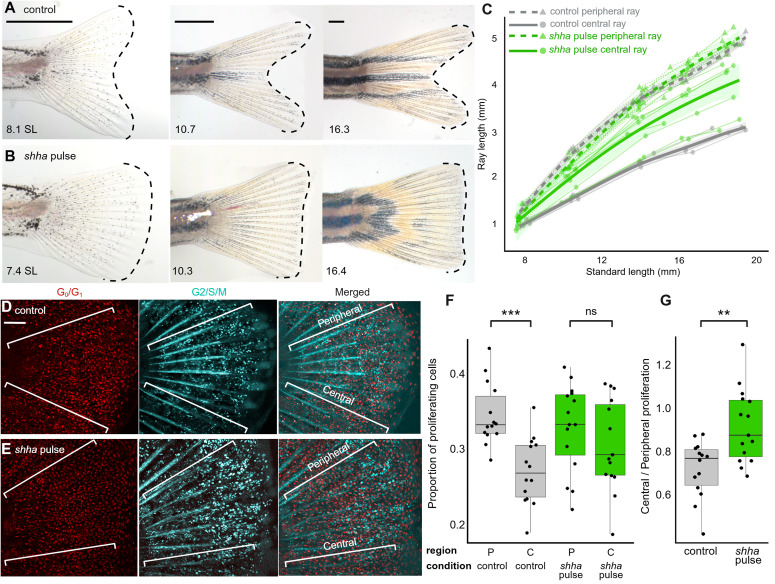
Divergent fin shape is accompanied by altered skeletal growth, presaged by differences in regional cell proliferation. **(A–B)** Individual fins imaged from 14 to 36 dpf, **(A)** non-transgenic WT control and **(B)** transgenic individual treated with *shha* pulse. Dashed lines indicate the distal edge and overall shape of the fins. Scale bars, 500 µm. **(C)** Repeated tracking of individuals (thin lines) throughout development shows the emergence of WT forked fin shape (gray lines) is the result of a lower growth rate in central rays (solid lines) relative to peripheral rays (dashed lines). Following embryonic *shha* pulse (green lines), central rays exhibit increased growth rates throughout development while peripheral rays retain a WT growth trajectory. (*n = *13 total larvae tracked) **(D–E)** Dual *z*-Fucci reporter showing non-proliferating cells (red) and cells in G2, S or M phase (cyan) in the dorsal lobe of caudal fin folds of **(D)** control larvae and **(E)** transgenic larvae treated with *shha* pulse. Scale bar, 100 µm. **(F)** In control developing forked fins, proliferation is relatively lower in central regions, while *shha* pulse causes increased proliferation in central regions of the developing truncate fin. Regions are indicated as peripheral (P) or central (C). Significance determined by linear mixed-effects model followed by Tukey’s post hoc test. **(G)** Across the entire organ, proliferation becomes more uniform (closer to 1) following *shha* pulse compared to controls developing forked fins. Significance determined by Welch two-tailed *t* test. The data underlying the graphs shown in the figure can be found in S1 Data and the summary statistics in S2 Data.

We inferred that the truncate phenotype induced by *shha* pulse involved a change in the growth rates between central and peripheral rays. In caudal fins of WT zebrafish, central rays grow more slowly than peripheral rays, causing the forked shape to become progressively pronounced as fish grow (Figs 5A, 5C, and S1D). In caudal fins of fish treated with *shha* pulse, peripheral rays grew at rates similar to those of WT controls (compare dashed lines in Fig 5C). In contrast, the central rays of truncate fins grew approximately 35% faster than central rays of controls, closer to the growth rates of the peripheral rays (compare solid lines in Fig 5B–5C).

We tested whether this acceleration of linear growth in the central rays following the *shha* pulse was driven by increased rates of local cell proliferation. We used the Dual *z*-Fucci transgenic line to report cell cycle states [68], using the line to quantify the proportion of all cells in G_2_/S/M phase in the peripheral regions compared to those in central regions of fins. During early larval stages of WT forked caudal fin development, peripheral and central regions possessed similar numbers of cells (S7A Fig), however differences in regional cell proliferation are already detectable, with fewer proliferative cells in central regions compared to peripheral regions (Fig 5D and 5F–5G; also see [32]). Peripheral regions ultimately accumulate more cells (S7A–S7B Fig) and exhibit faster growth rates (Fig 5C), creating the emerging forked shape. Cells in peripheral regions of fins treated with *shha* pulse showed rates of proliferation indistinguishable from those in peripheral regions of control fins (compare “P” regions in Figs 5F and S7). In contrast, the central regions of growing truncate fins showed elevated relative rates of proliferation (Fig 5G). These results suggest that relative rates of regional proliferation that will sculpt the fin shape are imprinted early in development, and can be disrupted by embryonic *shha* pulse.

Distal tips of juvenile fin rays have active Shh signaling during outgrowth [40,44], and *ptch2:kaede* [59] activity is enriched in peripheral rays compared to central rays, indicating relatively higher Shh pathway activity (S8 Fig; [44]). In contrast, fish treated with *shha* pulse showed relatively expanded expression of the *ptch2:kaede* reporter in central fin rays during juvenile outgrowth (S8 Fig).

### Fin shape imprinted during embryonic development informs regeneration and can be decoupled from length and proximodistal ray patterning

**Fig 6:**
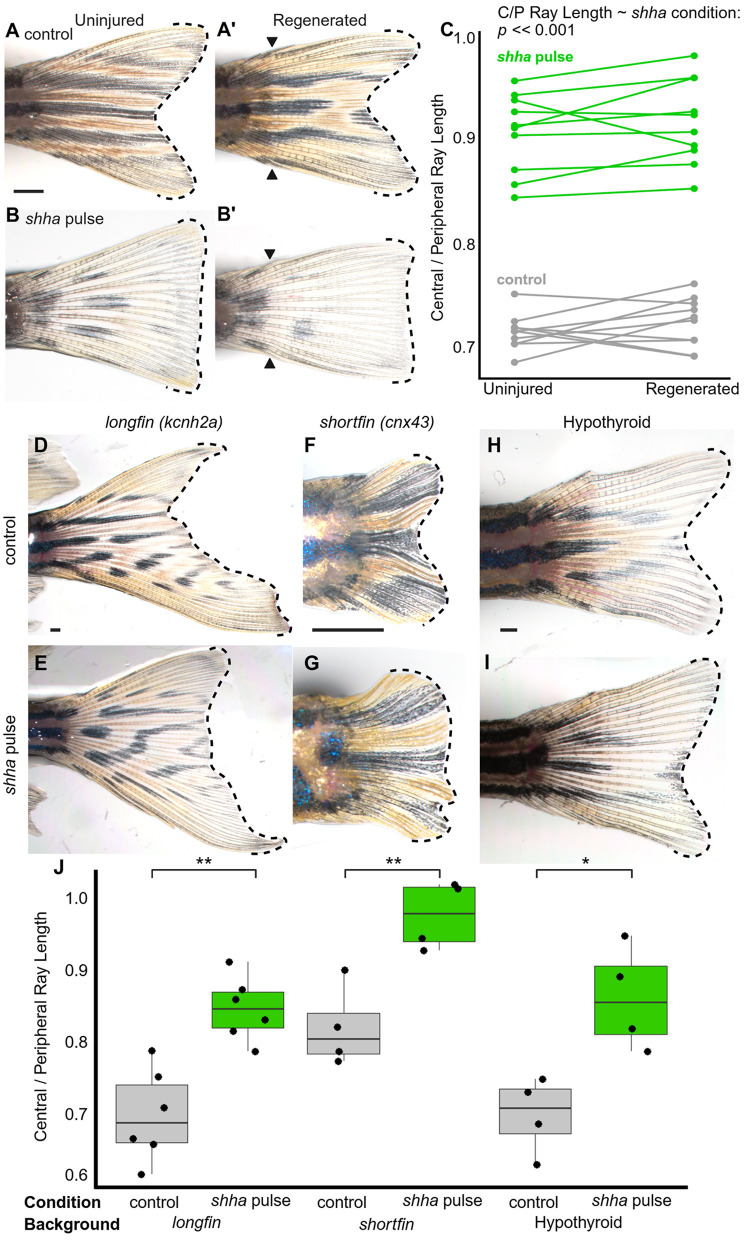
Imprinted caudal fin shape is remembered and may be decoupled from caudal fin length and proximodistal patterning. **(A–B)** Control WT caudal fins (**A**) restore a forked shape 30 days after amputation. **(B)** Truncate fins restore a truncate shape after amputation*.* (**A’, B’**) show fins (**A, B**) at 30 dpa, black arrowheads indicate amputation plane. Scale bar, 1 mm. **(C)** Quantification showing the fin shape of individuals before and after regeneration. Significance between factors determined via a linear mixed-effects model. **(D, F, H)** *longfin* mutants, *shortfin* mutants, and hypothyroid zebrafish all show forked fin shape. **(E, G, I)** *shha* pulse induces a truncate fin shape in all three backgrounds. **(J)** Quantification of fin shape in different backgrounds. Significance within each background is determined by Welch’s two-tailed *T*-tests. Scale bars, 500 µm. The data underlying the graphs shown in the figure can be found in S1 Data and the summary statistics in S2 Data.

Caudal fins regenerate to their original length and shape using remembered positional information (Fig 6A and 6C; [16,23,69]). We asked if the truncate fin shape was remembered and could inform regeneration following amputation. Indeed, truncate fins regenerated with truncate shapes (Fig 6B–6C), indicating that the information imprinted in the embryonic fin not only informs the development of fin shape, but also imprints long-term memory that is accessed during regeneration.

Numerous developmental or genetic alterations induce changes in fin morphology, including shifts in overall fin length or alterations in the skeletal patterning of rays along the proximodistal axis. Despite substantial shifts in morphology, these modified phenotypes maintain an overall forked caudal fin shape with short central rays (see [7,33,34,36,38] and Fig 6D, 6F and 6H). Noting that truncate fins are comparable in overall length and proximodistal patterning to WT forked fins, we predicted that these aspects of fin morphology were regulated by independent pathways that could be developmentally decoupled. We introduced *shha* pulse in mutant backgrounds with long (*lof*) or short (*sof*) fin lengths [33,34,36], as well as in a hypothyroid background that proximalizes fin ray patterning [38]. Consistent with the prediction, truncate fins could be induced even in lengthened, shortened or proximalized backgrounds (Fig 6D–6J), suggesting that overall length, proximo-distal position of bifurcation and relative length of central and peripheral rays are each regulated by independent signaling pathways.

## Discussion

Coordinated morphogenetic processes create complex and functional organ shapes on which natural selection can act. Positional identities may be imprinted at early developmental stages, and these remembered identities can govern cell behaviors and the emergence of shape during later morphogenesis. The transient activity of the Shh pathway specifies positional identity in multiple vertebrate contexts: in tetrapod limbs, the early activity of Shh imprints positional identities of digits in mammals [70] and flight feathers in birds [71]. Although Shh is not normally involved in imprinting or patterning the early caudal fin, we have shown that a transient pulse of Shh activity can alter the morphogenetic fates imprinted in the embryonic fin primordium, and that this is sufficient to re-pattern the shape into which the adult fin is fated to grow.

Limb buds and paired fins (as well as the dorsal and anal fins) all require Shh pathway activity to specify posterior identities [51,52,55,72]. Loss of Shh expression from the fins disrupts formation of both paired and dorsal fins [52,54,72], but ongoing or transient inhibition of the Shh pathway only minimally affects the external morphology of the caudal fin ([43,44,52,55]; also see BMS-treated control in Fig 1I). Although the Shh pathway is involved in later morphogenesis of the caudal fin rays [42–44], the early caudal fin primordium shows neither expression of *shha* or any other hedgehog family members [40,55], nor activity of a Shh pathway reporter [44]. Nonetheless, the early caudal fin primordium expresses downstream Shh effectors, including *gli3, smo, and ptch2* [40], likely sensitizing these tissues to a precocious *shha* pulse. Our data suggest a critical period extending through late embryogenesis during which the fate of the caudal fin shape is imprinted and remains sensitive to re-patterning. This period of sensitivity ends after 3 dpf (see Figs 2D and S5), well before fin rays are established [22] and before endogenous expression of *shha* in the caudal fin fold tissues (see Fig 2A; [40,55]).

The paired fins show distinct patterns of gene expression along the anteroposterior axis, with *alx4a* expressed specifically in anterior fin rays [73]. In the caudal fin, *alx4a* is expressed in the peripheral rays [17], but there is not corresponding central expression of posterior-identifying factors such as *hand2* [17,73]. Indeed, during different stages of ray outgrowth, several pathways are enriched at the growing tips of peripheral rays relative to central rays, including the Shh pathway itself ([44,55] and see S8 Fig), and nuclear thyroid hormone activity [38]. However, the increased activity of these pathways and the elevated expression of several transcripts in peripheral rays during homeostasis [25] and regeneration [45,69,74,75] may simply reflect the larger cell populations and higher metabolic demands of the faster-growing peripheral regions. Nonetheless, these pathways and genes represent candidates that may store or interpret positional information governing relative growth of rays.

The posterior of the body axis is patterned by progressively 5′ Hox genes [76]. The most posterior structure in a teleost is the caudal fin, which is regulated by *hox13* genes expressed in the posterior axis during the first week of development, before the emergence of any caudal fin skeletal elements (see Fig 4A and 4C; [7,64]). Disruption of 5′ Hox factors changes the patterning and development of the caudal fin [7,76]. In limbs and paired fins, Shh signaling functions in concert with 5′ Hox genes to establish anteroposterior patterning [49,67]. In tetrapod limbs, continuous Shh expression is required for maintenance and later expression of HoxA and D cluster genes [49], and Shh inhibits the repressor form of Gli3, which activates 5′ *HoxD* expression [77,78]. While HoxA and D are involved in the development of paired, dorsal and anal fins in both teleosts and chondrichthyans, these clusters do not appear to contribute to patterning in the caudal fin [79,80]. We found that the *shha* pulse led to expanded expression domains of *hox13* at 24 hrs and 3 days following treatment (Figs 4A–4D and S6). Given the known interactions between Shh and Hox in other contexts [66,70,81,82], there may be a direct induction of these Hox13 factors by Shh overactivity, and the shifts in Hox13 expression and overlap may be the proximate cause of the change in fin shape.

*hoxb13a* expression is normally restricted to the posterior-most tissues fated to become the dorsal lobe of the caudal fin [7,83]. Expanded expression domains–particularly the expanded expression of *hoxb13a*–and enlarged regions of overlapping expression between Hox13 factors may effectively “posteriorize” the regions that will give rise to the central hypurals and fin rays. Rays developing at the center of a fin exposed to *shha* pulse may adopt a more posterior identity, consequently adopting characteristics of longer, more peripheral rays. A central organizing center, producing as-yet-undiscovered morphogen(s), has been proposed to both establish the hypural diastema and specify the axis of symmetry and differential outgrowth properties of the progressively developing rays [17]. Such a signaling center may be established to imprint fin shape during the developmental period we identified, and a *shha* pulse may disrupt the organizing center, both blocking formation of the hypural diastema and allowing excessive growth in the central rays.

Relative rates of proliferation are frequently informed by positional context. Like the proportionally rapid proliferation of cells in the peripheral regions of a growing forked fin ([31,32] and see Fig 5D–5G), cells in the outer regions of an embryonic limb bud show the highest local rates of proliferation [84]. Our data show that shifts in regional proliferation in the larval fin correspond with the production of different adult fin shapes (Fig 5). The Shh pathway directly regulates proliferation in developing limbs [53,85–87]. However, the relative increase in central proliferation rates in developing truncate fins was not observed until approximately 10 days after the brief *shha* pulse was induced (Figs 5 and S7), so we posit that the relationship between excess *shha* and increased central proliferation is unlikely to be one of direct stimulation.

From the somitic paraxial mesoderm, the sclerotome contributes to the dorsal and anal median fins [80,88,89], and the migration of the sclerotome population can be regulated by the Shh pathway [90]. The occasional failure of the anal fin to form in fish treated with *shha* pulse (see S4 Fig) may be due to a sclerotome migration defect. However, the sclerotome is not a primary contributor to the caudal fin [89], and a potential sclerotome deficit seems unlikely to underlie the formation of the truncate caudal fin shape.

The shape of the caudal fin is restored following amputation, indicating that information directing the emergent shape is remembered and redeployed during regeneration. *shha* pulse, induced at a specific period of embryonic development, re-patterns caudal fin shape from forked to truncate, and this truncate shape is remembered and restored through regeneration (see Fig 6). The remembered overall length of the caudal fin can be overridden and reprogrammed by inhibiting proliferation during blastema formation at the onset of regeneration [45]. In contrast, *shha* pulse treatment occurs early in development to re-pattern the overall shape of the organ. This suggests that the positional information that will inform relative regional growth both during development and regeneration of the caudal fin is imprinted during a critical period of embryonic fin specification.

Alterations in calcineurin or thyroid hormone signaling during regeneration can shift the overall length or proximodistal patterning of a regenerating fin without changing the underlying memory, such that subsequent rounds of regeneration revert to a WT morphology [26,38,74]. Notably, the overall shape of the organ tends to remain forked through these treatments. We found that a *shha* pulse was capable of inducing excess central ray growth and a truncate shape in shortened, lengthened and proximalized backgrounds (Fig 6D–6J). These results suggest that fin shape is regulated independently from the pathways governing length and ray proximodistal patterning, and that individually modifying these developmental processes can effectively produce a wide range of fin phenotypes that phenocopy some of the natural diversity observed across modern teleosts (see Fig 6D–6J).

The hypural diastema, a space between hypurals 2 and 3, is considered a teleostean novelty [19], which was convergently acquired in gars [4]. The diastema has been independently lost at least once in most crown teleost lineages, including cusk, swamp and true eels and in derived groups of bony tongues, catfish, cod, flatfish and killifish [4,6,19,91,92]. Many of the clades that have lost a hypural diastema (e.g., killifish, flatfish) also grow with a truncate or rounded caudal fin shape [6,91], but the evolutionary relationship between the presence of a diastema and the shape of the fin has not been systematically explored. Previous work demonstrated that the Shh pathway is involved in the morphogenesis of the hypural complex: continuous overactivity of the Shh pathway expands hypural 2 [55]. Uniquely, however, fish treated with the brief *shha* pulse consistently lacked a hypural diastema (Fig 1G). We note that other zebrafish mutants with hypural defects or lacking a hypural diastema still develop with external forked shapes (see [7,55,93]), so we do not believe that the diastema its self informs the forked morphology.

The evolution of externally-symmetrical homocercal caudal fins in teleosts allowed the external skeleton to take on distinct dorsoventral functionalization [4,5,94,95]. This morphological functionalization likely supported the diversification of caudal fin shapes across teleosts. Truncate and forked fins each offer biomechanical advantages and tradeoffs as propulsive and stabilizing organs [4,9,10,96]. We have shown that an early *shha* pulse can induce a truncate shape, such that the external fin of a zebrafish resembles that of a medaka or killifish, and likely altering the hydrodynamic properties of the organ. Notably, the pigment pattern in the induced truncate zebrafish caudal fin (e.g., see Fig 5B) resembles the vertically-oriented arches or bars in several species with evolved truncate fins [97–99].

Our work identifies a critical window of embryonic development during which the positional information that will establish the adult fin shape is imprinted. We demonstrate that this positional information is independent from the developmental processes that regulate fin length and ray patterning along the proximodistal axis. Transient, precocious activation of the Shh pathway was sufficient to both abolish the hypural diastema and re-pattern the shape of the external caudal fin. In all, our findings suggest developmental mechanisms that may underlie natural teleost fin diversity, and can facilitate discovery of other mechanisms that imprint and deploy positional identity.

## Materials and methods

### Animal husbandry

All experiments with zebrafish were done in accordance with protocols approved by the Boston College Institutional Animal Care and Use Committee (protocol number 2007-006-01). Zebrafish were reared under standard conditions at 28 °C with a 14:10 light:dark cycle. Fish were fed marine rotifers, *Artemia*, Adult Zebrafish Diet (Zeigler, Gardners PA, USA) and Gemma Micro (Skretting, Stavanger, NOR).

For developmental serial imaging, fish from single clutches were reared in separate containers so individuals could be identified. Certain treatments (e.g., the *shha* pulse, below) caused changes in early growth rate, and we took care to size-match treated and control individuals; standard length (SL) is reported throughout. Note that prior to development of the hypural complex, notochord length was measured, and is referred to as SL, per [22].

### Transgenic and mutant lines

Transgenic lines used were *Tg*(*hsp70l:shha-EGFP*) [56] to induce *shha* pulse (below), *Tg(sp7:GFP)b1212* [100] to visualize osteoblasts, *Tg(p7.2sox10:mRFP)* [101] to visualize chondrocytes, *TgBAC(ptch2:Kaede)* [59] to visualize Shh-active cells and Dual *z*-Fucci [68,102] to identify cells in different phases of the cell cycle. Mutants used were *longfin*^*dt2*^*/kcnh2a* [34,103], and *shortfin*^*dj7e2*^*/cnx43* [33].

### Imaging

Zebrafish were anesthetized with tricaine (MS-222, approximately 0.02% w/v in fish system water). Cleared and stained [104] or anesthetized individuals were imaged on an Olympus SZX16 stereoscope using an Olympus DP74 camera, an Olympus IX83 inverted microscope using a Hamamatsu ORCA Flash 4.0 camera, a Leica Thunder Imager Model Organism using a sCMOS monochrome camera, or a Zeiss AxioImager Z2 using a Hamamatsu Flash4.0 V3 sCMOS camera. Identical exposure times and settings were used to compare experimental treatments and capture repeated images of fins. Images were correspondingly adjusted for contrast, brightness and color balance using FIJI [105], and compiled using BioRender.

### *shha* pulse

To induce a pulse of *shha* overexpression, clutches of *Tg*(*hsp70l:shha-EGFP*) were treated with 37 °C heat shock for 15 min, at 2 dpf (unless otherwise noted for experiment). Individuals were sorted for GFP expression following heat shock as in [56]. Clutch-mate larvae that were treated with heat shock but were negative for GFP were kept as the WT negative controls. Typically, embryos with the brightest GFP were kept as the *shha* pulse group (unless otherwise noted for experiment).

### Local *shha* overexpression

Local activation of the heat shock promoter was performed as previously described [63]. Local heat shocks were induced in clutches at 2 dpf for 15 min; local GFP expression was confirmed 16–18 hrs after the treatment. Embryos in which GFP was not detectable were either negative for the transgene or the heat and duration were not sufficient to induce expression; the genotypes of this control group were not tested. For sham heat shocks, larvae were touched at the posterior notochord with a cold probe for 15 min.

### Ray length measurements and amputations

On WT and control fins, the 2nd dorsal ray measured as the “peripheral ray” and the 9th dorsal ray was measured as the “central ray”. Individuals treated with *shha* pulse often developed fewer than 18 principal rays, so the central-most ray was measured as the central ray. Ray lengths were measured using segmented lines in FIJI [105].

Adult caudal fin regeneration experiments were performed on adult zebrafish >27 mm SL. Caudal fins were amputated from anesthetized fish under a stereoscope at the 5th proximal ray segment using a razor blade.

### Drug treatments

To rescue the *shha* overexpression phenotype by Shh pathway inhibition, larvae were treated either with the Smoothened inhibitor BMS-833923 [58] (0.5 µM + 0.05% DMSO in fish water) or the vehicle control DMSO (0.05% in fish water). A clutch of *Tg*(*hsp70l:shha-eGFP*) was treated with heat shock and sorted as above, and transgenic (GFP+) and non-transgenic (GFP-). Groups were treated with either BMS or the vehicle control for 4 hrs starting 16–18 hrs after heat shock (3 dpf). The treatment was repeated a second time 24 hrs after the first treatment (4 dpf). After washout, fish were reared to adulthood under standard conditions.

To induce hypothyroidism during regeneration, fins were amputated as above, and fish were treated with MPI cocktail (1.0 mM MMI + 0.1 mM KClO_4_ + 0.01 mM iopanoic acid, diluted in fish water) [38,99], for 21 days with drug changes every 1–2 days.

### Quantitative PCR

Controls and *shha* pulse-treated larvae were sampled at specific time points with *n* = 5 larvae placed into Thermo Fisher RNAlater Stabilization Solution (Cat. #: AM7021). RNA was extracted using Zymo Research Quick-RNA Microprep Kit (Cat. #: R1050), gDNA was degraded using ThermoFisher ezDNase Enzyme (Cat. #: 11766051) and cDNA libraries synthetized using Thermo Fisher SuperScript IV Reverse Transcriptase (Cat. #: 18090010). For the qPCR to assess genomic *gfp* copy number, dorsal fins were collected from individual adults and gDNA was isolated by phenol extraction. Thermo Fisher PowerUp SYBR Green Master Mix (Cat. #: A25741) was used for all qPCR, and primers are listed in S1 Table. Three technical replicates and three biological replicates were run on Thermo Fisher QuantStudio 3 Real-Time PCR System (Cat. #: A28567), and results were analyzed using the ThermoFisher Connect Platform and RStudio.

### Whole mount fluorescent *in situ* hybridization

Fish were anesthetized, fixed for 30 min in 4% PFA, and dehydrated then rehydrated in a methanol series. Fish were stained as described [106], with the modification that all 0.2× SSCT washes were only performed twice. RNAscope Multiplex Fluorescent Reagent Kit v2 (ACD Bio-techne, 323100) was used to target four *hox13* genes (ACD Bio-techne: *hoxa13b* 1129201-C2, *hoxb13a* 882111-C1, *hoxc13a* 1690451-C4, and *hoxd13a* 119137-C4).

### Proliferation quantification

Proliferation was measured in different regions of the growing fins using the Dual *z*-Fucci transgenic line [68]. Four rays were measured from each fin: the 2nd dorsal and 2nd ventral rays (the peripheral rays) and the two center-most rays of each lobe (the central rays). Proliferation was measured along a segmented line drawn through the center of each ray, and was calculated as the number of cyan cells divided by the total number of cyan plus red cells. For each individual, the regional proliferation was calculated as the average proliferation of the two peripheral rays and the average proliferation of the two central rays.

### Statistical analysis

Analyses were performed in RStudio. Data were analyzed with Welch two-sample, two-tailed *t* test, ANOVA followed by Tukey’s honest significant differences (using 95% family-wise confidence level), Fligner-Killeen test, or linear mixed-effects model (followed by Tukey’s honest significant differences using 95% family-wise confidence level). In graphs showing pairwise comparisons, significance is indicated as follows: * *p* < 0.05, ** *p* < 0.01, *** *p* < 0.001. Each plotted data point represents a single measurement from a single fish, unless otherwise noted in the figure legend.

## Supporting information

S1 Fig Growth of body and fins under different *shha* profiles. **(A–B)** Whole body images of **(A)** control and **(B)** transgenic clutch mates treated with *shha* pulse. Scale bars, 500 µm. **(C)** The overall length of the caudal fin (as measured by the length of the peripheral 2nd dorsal ray) relative to the standard length (SL), tracked in individuals from the same clutch for the first 6 weeks of development. By 30 dpf, truncate fins were the same length as the forked fins of controls. **(D)** The difference in caudal fin shape between conditions is evident by 14 dpf. Significance within each time point determined by Welch’s two-tailed *T*-tests. The data underlying the graphs shown in the figure can be found in S1 Data and the summary statistics in S2 Data.
(TIF)

S2 Fig Caudal fin shape shows no interaction with sex. Representative caudal fins of male and female **(A)** control and **(B)** *shh* pulse-treated individuals. Scale bar, 1 mm. **(C)** There was no difference in fin shape between sexes in either control or *shh* pulse-treated fish. Significance determined by ANOVA followed by Tukey’s post hoc test. The data underlying the graphs shown in the figure can be found in S1 Data and the summary statistics in S2 Data.
(TIF)

S3 Fig *shha* pulse does not affect shape of paired, dorsal or anal fins. Shapes of fins were quantified as the ratio in lengths of the shortest bifurcating ray to the longest bifurcating ray. Adult dorsal (***A–B***), anal (***C–D***), pectoral (***E–F***) and pelvic fins (***G–H***) showed no differences in shape after *shha* pulse compared to control clutch mates. Scale bars, 1 mm. Significance determined using Welch’s two-tailed *T*-tests. The data underlying the graphs shown in the figure can be found in S1 Data and the summary statistics in S2 Data.
(TIF)

S4 Fig *shha* pulse occasionally disrupts anal fin development. (***A***) In approximately 75% of individuals treated with *shha* pulse, the anal fin develops with a shape and size comparable to that those in control individuals. (***B***) In approximately 20% of fish treated with *shha* pulse, no anal fin develops. **(C–D)** In approximately 5% of individuals treated with *shha* pulse, a reduced number of endoskeletal bones (black arrow) and fin rays develop. Scale bar, 1 mm. Phenotype percentages displayed in (***E***).
(TIF)

S5 Fig *shha* pulse induction after 3 days post fertilization does not induce truncate fin shapes despite effectively producing transgene overexpression. (***A***) Caudal fins of fish treated with *shha* pulse at different days post-fertilization. Dashed outlines indicate the overall shape of the fins. Scale bar, 500 µm. (***B***) Individuals were heat shocked at different days post-fertilization, and mean fluorescence intensity was measured 16–18 hrs later. Each data point represents an individual larva. GFP was visible in transgenic larvae after each heat shock treatment, regardless of day of heat shock. (***C–D***) Individuals were heat-shocked at different days post-fertilization and expression of *shha* (***C***) and *ptch2* (***D***) were measured by qRT-PCR. Each datapoint represents a biological replicate of 3 pooled larvae, collected at approximately 6 hrs after heat shock, normalized to a single replicate in the control group. Significance within each time point determined by Welch’s two-tailed *T*-tests, and the correlation between the conditional readout and age at heat shock determined by linear-mixed effects model. The data underlying the graphs shown in the figure can be found in S1 Data and the summary statistics in S2 Data.
(TIF)

S6 Fig *hox13* expression during early larval caudal fin development responds to *shha* pulse. Caudal fins of WT controls and individuals treated with *shha* pulse caudal fin folds at 3 and 5 days post-fertilization. Merged images of **hoxa13b* *+ *hoxd13a* and **hoxb13a* *+ *hoxc13a* are displayed next to the single-channel images. A minimum of 3 individuals were examined for each condition and time point. Small white arrows indicate the posterior end of the caudal artery. Standard lengths reported are corrected after fixation [19]. Scale bar, 100 µm.
(TIF)

S7 Fig *shha* pulse induces larger cell populations in central regions of fins. (***A***) At earlier stages of larval development (5.8–6.5 SL) there is no difference in cell number between central and peripheral fin regions in either condition (non-transgenic control or *shha* pulse). (***B***) At later stages of larval development (6.5–7.2 SL), control larvae show relatively fewer cells in central regions, but larvae treated with *shha* pulse show similar cell numbers in central and peripheral regions. (***C***) Dorsal and ventral regions of developing larval fins do not show different proportions of proliferating cells. Significance determined by linear mixed-effects model followed by Tukey’s post hoc test; statistically indistinguishable groups are shown with the same letter (threshold for significance *p* < 0.05). The data underlying the graphs shown in the figure can be found in S1 Data and the summary statistics in S2 Data.
(TIF)

S8 Fig Size of *ptch2.kaede* activity domain correlates with relative ray length. **(A–B)** Repeated tracking of individual *Tg(ptch2:kaede)* larvae during early caudal fin development. Image series performed on a minimum of 7 larvae per condition. Distal domains of expression in peripheral and central rays utilized for quantification in **(C)** shown in brackets. Scale bar, 100 µm. **(C)** The domain length ratio of the central to the peripheral region between conditions. Significance determined using Welch’s two-tailed *t* test. The data underlying the graphs shown in the figure can be found in S1 Data and the summary statistics in S2 Data.
(TIF)

S1 Table Primer sets used for qPCR and RT-qPCR. (DOCX)

S1 Data Raw data. (XLSX)

S2 Data Summary statistics. (XLSX)

## Abbreviations

**:**
dpf: days post-fertilization
Shh: sonic hedgehog
SL: standard length
WT: wild-type
ZPA: zone of polarizing activity

## References

[1] Sears K, Maier JA, Sadier A, Sorensen D, Urban DJ. Timing the developmental origins of mammalian limb diversity. Genesis. 2018;56(1). doi: 10.1002/dvg.23079 29095555

[2] Wolpert L. Positional information and pattern formation in development. Dev Genet. 1994;15(6):485–90. doi: 10.1002/dvg.1020150607 7834908

[3] Polly PD. Chapter 15 - Limbs in mammalian evolution. In: Hall BK, editor. Fins into limbs: evolution, development, and transformation. Chicago: University of Chicago Press; 2007. p. 245–68.

[4] Desvignes T, Carey A, Postlethwait JH. Evolution of caudal fin ray development and caudal fin hypural diastema complex in spotted gar, teleosts, and other neopterygian fishes. Dev Dyn. 2018;247(6):832–53. doi: 10.1002/dvdy.24630 29569346 PMC5980753

[5] Lauder GV. Caudal fin locomotion in ray-finned fishes: historical and functional analyses. Am Zool. 1989;29(1):85–102.

[6] Arratia G. Complexities of early teleostei and the evolution of particular morphological structures through time. Copeia. 2015;103(4):999–1025. doi: 10.1643/cg-14-184

[7] Cumplido N, Arratia G, Desvignes T, Muñoz-Sánchez S, Postlethwait JH, Allende ML. Hox genes control homocercal caudal fin development and evolution. Sci Adv. 2024;10(3):eadj5991. doi: 10.1126/sciadv.adj5991 38241378 PMC10798566

[8] Webb PW. Body form, locomotion and foraging in aquatic vertebrates. Am Zool. 1984;24(1):107–20.

[9] Tack NB, Gemmell BJ. A tale of two fish tails: does a forked tail really perform better than a truncate tail when cruising? J Exp Biol. 2022;225(22):jeb244967. doi: 10.1242/jeb.244967 36354328

[10] Flammang BE, Lauder GV. Caudal fin shape modulation and control during acceleration, braking and backing maneuvers in bluegill sunfish, *Lepomis macrochirus*. J Exp Biol. 2009;212(Pt 2):277–86. doi: 10.1242/jeb.021360 19112147

[11] Giammona FF. Form and function of the caudal fin throughout the phylogeny of fishes. Integr Comp Biol. 2021;61(2):550–72. doi: 10.1093/icb/icab127 34114010

[12] Flammang BE. The fish tail as a derivation from axial musculoskeletal anatomy: an integrative analysis of functional morphology. Zoology (Jena). 2014;117(1):86–92. doi: 10.1016/j.zool.2013.10.001 24290784

[13] Liao JC. Fish swimming efficiency. Curr Biol. 2022;32(12):R666–71. doi: 10.1016/j.cub.2022.04.073 35728550

[14] Pfefferli C, Jaźwińska A. The art of fin regeneration in zebrafish. Regeneration. 2015;2(2):72–83. 27499869 10.1002/reg2.33 PMC4895310

[15] Henke K, Farmer DT, Niu X, Kraus JM, Galloway JL, Youngstrom DW. Genetically engineered zebrafish as models of skeletal development and regeneration. Bone. 2023;167:116611. 36395960 10.1016/j.bone.2022.116611 PMC11080330

[16] Harris MP, Daane JM, Lanni J. Through veiled mirrors: fish fins giving insight into size regulation. Wiley Interdiscip Rev Dev Biol. 2021;10(4):e381. doi: 10.1002/wdev.381 32323915

[17] Desvignes T, Robbins AE, Carey AZ, Bailon-Zambrano R, Nichols JT, Postlethwait JH, et al. Coordinated patterning of zebrafish caudal fin symmetry by a central and two peripheral organizers. Dev Dyn. 2022;251(8):1306–21. doi: 10.1002/dvdy.475 35403297 PMC9357109

[18] Sehring IM, Weidinger G. Recent advancements in understanding fin regeneration in zebrafish. Wiley Interdiscip Rev Dev Biol. 2020;9(1):e367. doi: 10.1002/wdev.367 31726486

[19] Schultze HP, Arratia G. The caudal skeleton of basal teleosts, its conventions, and some of its major evolutionary novelties in a temporal dimension. Mesozoic Fishes 5 – Global Diversity and Evolution. 2013. p. 187–246.

[20] Cumplido N, Allende ML, Arratia G. From devo to evo: patterning, fusion and evolution of the zebrafish terminal vertebra. Front Zool. 2020;17:18. doi: 10.1186/s12983-020-00364-y 32514281 PMC7268543

[21] Sanger TJ, McCune AR. Comparative osteology of the Danio (Cyprinidae: Ostariophysi) axial skeleton with comments on Danio relationships based on molecules and morphology. Zool J Linn Soc. 2002;135(4):529–46.

[22] Parichy DM, Elizondo MR, Mills MG, Gordon TN, Engeszer RE. Normal table of postembryonic zebrafish development: staging by externally visible anatomy of the living fish. Dev Dyn. 2009;238(12):2975–3015. doi: 10.1002/dvdy.22113 19891001 PMC3030279

[23] Uemoto T, Abe G, Tamura K. Regrowth of zebrafish caudal fin regeneration is determined by the amputated length. Sci Rep. 2020;10(1):649. doi: 10.1038/s41598-020-57533-6 31959817 PMC6971026

[24] Azevedo AS, Grotek B, Jacinto A, Weidinger G, Saúde L. The regenerative capacity of the zebrafish caudal fin is not affected by repeated amputations. PLoS One. 2011;6(7):e22820. doi: 10.1371/journal.pone.0022820 21829525 PMC3145768

[25] Rabinowitz JS, Robitaille AM, Wang Y, Ray CA, Thummel R, Gu H, et al. Transcriptomic, proteomic, and metabolomic landscape of positional memory in the caudal fin of zebrafish. Proc Natl Acad Sci U S A. 2017;114(5):E717–26. doi: 10.1073/pnas.1620755114 28096348 PMC5293114

[26] Daane JM, Lanni J, Rothenberg I, Seebohm G, Higdon CW, Johnson SL, et al. Bioelectric-calcineurin signaling module regulates allometric growth and size of the zebrafish fin. Sci Rep. 2018;8(1):10391. doi: 10.1038/s41598-018-28450-6 29991812 PMC6039437

[27] Wehner D, Weidinger G. Signaling networks organizing regenerative growth of the zebrafish fin. Trends Genet. 2015;31(6):336–43. doi: 10.1016/j.tig.2015.03.012 25929514

[28] Benard EL, Küçükaylak I, Hatzold J, Berendes KUW, Carney TJ, Beleggia F. Wnt10a is required for zebrafish median fin fold maintenance and adult unpaired fin metamorphosis. Dev Dyn. 2023. 37870737 10.1002/dvdy.672 PMC11035493

[29] Kujawski S, Lin W, Kitte F, Börmel M, Fuchs S, Arulmozhivarman G, et al. Calcineurin regulates coordinated outgrowth of zebrafish regenerating fins. Dev Cell. 2014;28(5):573–87. 24561038 10.1016/j.devcel.2014.01.019

[30] Sakaguchi S, Nakatani Y, Takamatsu N, Hori H, Kawakami A, Inohaya K. Medaka unextended-fin mutants suggest a role for Hoxb8a in cell migration and osteoblast differentiation during appendage formation. Dev Biol. 2006;293(2):426–38. doi: 10.1016/j.ydbio.2006.02.022 16546159

[31] Goldsmith MI, Fisher S, Waterman R, Johnson SL. Saltatory control of isometric growth in the zebrafish caudal fin is disrupted in long fin and rapunzel mutants. Dev Biol. 2003;259(2):303–17. doi: 10.1016/s0012-1606(03)00186-6 12871703

[32] Jain I, Stroka C, Yan J, Huang W-M, Iovine MK. Bone growth in zebrafish fins occurs via multiple pulses of cell proliferation. Dev Dyn. 2007;236(9):2668–74. doi: 10.1002/dvdy.21270 17676636

[33] Perathoner S, Daane JM, Henrion U, Seebohm G, Higdon CW, Johnson SL, et al. Bioelectric signaling regulates size in zebrafish fins. PLoS Genet. 2014;10(1):e1004080. doi: 10.1371/journal.pgen.1004080 24453984 PMC3894163

[34] Stewart S, Le Bleu HK, Yette GA, Henner AL, Robbins AE, Braunstein JA, et al. Longfin causes cis-ectopic expression of the kcnh2a ether-a-go-go K+ channel to autonomously prolong fin outgrowth. Development. 2021;148(11):dev199384. doi: 10.1242/dev.199384 34061172 PMC8217709

[35] Iovine MK, Higgins EP, Hindes A, Coblitz B, Johnson SL. Mutations in connexin43 (GJA1) perturb bone growth in zebrafish fins. Dev Biol. 2005;278(1):208–19. 15649473 10.1016/j.ydbio.2004.11.005

[36] Hoptak-Solga AD, Klein KA, DeRosa AM, White TW, Iovine MK. Zebrafish short fin mutations in connexin43 lead to aberrant gap junctional intercellular communication. FEBS Lett. 2007;581(17):3297–302. doi: 10.1016/j.febslet.2007.06.030 17599838 PMC2044562

[37] Silic MR, Wu Q, Kim BH, Golling G, Chen KH, Freitas R, et al. Potassium channel-associated bioelectricity of the dermomyotome determines fin patterning in zebrafish. Genetics. 2020;215(4):1067–84. doi: 10.1534/genetics.120.303390 32546498 PMC7404225

[38] Harper M, Hu Y, Donahue J, Acosta B, Dievenich Braes F, Nguyen S, et al. Thyroid hormone regulates proximodistal patterning in fin rays. Proc Natl Acad Sci U S A. 2023;120(21):e2219770120. doi: 10.1073/pnas.2219770120 37186843 PMC10214145

[39] Cardeira-da-Silva J, Bensimon-Brito A, Tarasco M, Brandão AS, Rosa JT, Borbinha J, et al. Fin ray branching is defined by TRAP+ osteolytic tubules in zebrafish. Proc Natl Acad Sci U S A. 2022;119(48):e2209231119. doi: 10.1073/pnas.2209231119 36417434 PMC9889879

[40] Hadzhiev Y, Lele Z, Schindler S, Wilson SW, Ahlberg P, Strähle U, et al. Hedgehog signaling patterns the outgrowth of unpaired skeletal appendages in zebrafish. BMC Dev Biol. 2007;7:75. doi: 10.1186/1471-213X-7-75 17597528 PMC1950712

[41] Laforest L, Brown CW, Poleo G, Géraudie J, Tada M, Ekker M, et al. Involvement of the sonic hedgehog, patched 1 and bmp2 genes in patterning of the zebrafish dermal fin rays. Development. 1998;125(21):4175–84. doi: 10.1242/dev.125.21.4175 9753672

[42] Quint E, Smith A, Avaron F, Laforest L, Miles J, Gaffield W. Bone patterning is altered in the regenerating zebrafish caudal fin after ectopic expression of sonic hedgehog and bmp2b or exposure to cyclopamine. Proc Natl Acad Sci. 2002;99(13):8713–8. doi: 10.1073/pnas.132258999 12060710 PMC124364

[43] Armstrong BE, Henner A, Stewart S, Stankunas K. Shh promotes direct interactions between epidermal cells and osteoblast progenitors to shape regenerated zebrafish bone. Development. 2017;144(7):1165–76. 28351866 10.1242/dev.143792 PMC5399624

[44] Braunstein JA, Robbins AE, Stewart S, Stankunas K. Basal epidermis collective migration and local sonic hedgehog signaling promote skeletal branching morphogenesis in zebrafish fins. Dev Biol. 2021;477:177–90. 34038742 10.1016/j.ydbio.2021.04.010 PMC10802891

[45] Wang YT, Tseng TL, Kuo YC, Yu JK, Su YH, Poss KD. Genetic reprogramming of positional memory in a regenerating appendage. Curr Biol. 2019;29(24):4193-4207.e4. doi: 10.1016/j.cub.2019.11.018 PMC6917923 31786062

[46] Lettice LA, Heaney SJH, Purdie LA, Li L, de Beer P, Oostra BA, et al. A long-range Shh enhancer regulates expression in the developing limb and fin and is associated with preaxial polydactyly. Hum Mol Genet. 2003;12(14):1725–35. doi: 10.1093/hmg/ddg180 12837695

[47] Riddle RD, Johnson RL, Laufer E, Tabin C. Sonic hedgehog mediates the polarizing activity of the ZPA. Cell. 1993;75(7):1401–16. 8269518 10.1016/0092-8674(93)90626-2

[48] Sagai T, Masuya H, Tamura M, Shimizu K, Yada Y, Wakana S, et al. Phylogenetic conservation of a limb-specific, cis-acting regulator of sonic hedgehog (Shh). Mamm Genome. 2004;15(1):23–34. doi: 10.1007/s00335-033-2317-5 14727139

[49] Chiang C, Litingtung Y, Harris MP, Simandl BK, Li Y, Beachy PA. Manifestation of the limb prepattern: limb development in the absence of sonic hedgehog function. Dev Biol. 2001;236(2):421–35. 10.1006/dbio.2001.0346 11476582

[50] Dahn RD, Davis MC, Pappano WN, Shubin NH. Sonic hedgehog function in chondrichthyan fins and the evolution of appendage patterning. Nature. 2007;445(7125):311–4. doi: 10.1038/nature05436 17187056

[51] Onimaru K, Kuraku S, Takagi W, Hyodo S, Sharpe J, Tanaka M. A shift in anterior–posterior positional information underlies the fin-to-limb evolution. eLife. 2015;4:e07048. doi: 10.7554/eLife.07048 26283004 PMC4538735

[52] Letelier J, de la Calle-Mustienes E, Pieretti J, Naranjo S, Maeso I, Nakamura T, et al. A conserved Shh cis-regulatory module highlights a common developmental origin of unpaired and paired fins. Nat Genet. 2018;50(4):504–9. doi: 10.1038/s41588-018-0080-5 29556077 PMC5896732

[53] Letelier J, Naranjo S, Sospedra-Arrufat I, Martinez-Morales JR, Lopez-Rios J, Shubin N, et al. The Shh/Gli3 gene regulatory network precedes the origin of paired fins and reveals the deep homology between distal fins and digits. Proc Natl Acad Sci U S A. 2021;118(46):e2100575118. doi: 10.1073/pnas.2100575118 34750251 PMC8673081

[54] Hawkins MB, Jandzik D, Tulenko FJ, Cass AN, Nakamura T, Shubin NH. An Fgf–Shh positive feedback loop drives growth in developing unpaired fins. Proc Natl Acad Sci. 2022;119(10):e2120150119. doi: 10.1073/pnas.2120150119 35238632 PMC8916008

[55] Tanaka Y, Okayama S, Urakawa K, Kudoh H, Ansai S, Abe G. Fin elaboration via anterior-posterior constraint by hhip on hedgehog signaling in teleosts. Dev Camb Engl. 2024. doi: dev.202526 10.1242/dev.202526 PMC11607692 39417578

[56] Shen M-C, Ozacar AT, Osgood M, Boeras C, Pink J, Thomas J, et al. Heat-shock-mediated conditional regulation of hedgehog/gli signaling in zebrafish. Dev Dyn. 2013;242(5):539–49. doi: 10.1002/dvdy.23955 23441066

[57] Arveseth CD, Happ JT, Hedeen DS, Zhu JF, Capener JL, Shaw DK. Smoothened transduces Hedgehog signals via activity-dependent sequestration of PKA catalytic subunits. PLOS Biol. 2021;19(4):e3001191. doi: 10.1371/journal.pbio.3001191 33886552 PMC8096101

[58] Lin TL, Matsui W. Hedgehog pathway as a drug target: Smoothened inhibitors in development. Onco Targets Ther. 2012;5:47–58. doi: 10.2147/OTT.S21957 22500124 PMC3325001

[59] Huang P, Xiong F, Megason SG, Schier AF. Attenuation of notch and Hedgehog signaling is required for fate specification in the spinal cord. PLoS Genet. 2012;8(6):e1002762. doi: 10.1371/journal.pgen.1002762 22685423 PMC3369957

[60] Towers M, Signolet J, Sherman A, Sang H, Tickle C. Insights into bird wing evolution and digit specification from polarizing region fate maps. Nat Commun. 2011;2:426. doi: 10.1038/ncomms1437 21829188

[61] Pickering J, Towers M. Inhibition of Shh signalling in the chick wing gives insights into digit patterning and evolution. Development. 2016;143(19):3514–21. doi: 10.1242/dev.137398 27702785 PMC5087615

[62] Smith M, Hickman A, Amanze D, Lumsden A, Thorogood P. Trunk neural crest origin of caudal fin mesenchyme in the zebrafish *Brachydanio rerio*. Proc R Soc Lond B Biol Sci. 1997;256(1346):137–45.

[63] Placinta M, Shen M-C, Achermann M, Karlstrom RO. A laser pointer driven microheater for precise local heating and conditional gene regulation in vivo. Microheater driven gene regulation in zebrafish. BMC Dev Biol. 2009;9:73. doi: 10.1186/1471-213X-9-73 20042114 PMC2810295

[64] Ye Z, Kimelman D. Hox13 genes are required for mesoderm formation and axis elongation during early zebrafish development. Development. 2020;147(22):dev185298. doi: 10.1242/dev.185298 33154036 PMC7710019

[65] Ahn D, Ho RK. Tri-phasic expression of posterior Hox genes during development of pectoral fins in zebrafish: implications for the evolution of vertebrate paired appendages. Dev Biol. 2008;322(1):220–33. 10.1016/j.ydbio.2008.06.032 18638469

[66] Ishizaka M, Maeno A, Nakazawa H, Fujii R, Oikawa S, Tani T. The functional roles of zebrafish HoxA- and HoxD-related clusters in the pectoral fin development. Sci Rep. 2024;14(1):23602. 10.1038/s41598-024-74134-9 39384796 PMC11464670

[67] Zákány J, Kmita M, Duboule D. A dual role for Hox genes in limb anterior-posterior asymmetry. Science. 2004;304(5677):1669–72. 10.1126/science.1096049 15192229

[68] Bouldin CM, Kimelman D. Dual fucci: a new transgenic line for studying the cell cycle from embryos to adults. Zebrafish. 2014;11(2):182–3. doi: 10.1089/zeb.2014.0986 24661087 PMC4518878

[69] Rich A, Lu Z, Simone AD, Garcia L, Janssen J, Ando K, et al. Decaying and expanding Erk gradients process memory of skeletal size during zebrafish fin regeneration [Internet]. bioRxiv; 2025 [cited 2025 Jan 28]. p. 2025.01.23.634576. Available from: https://www.biorxiv.org/content/10.1101/2025.01.23.634576v1 10.1038/s41567-026-03426-w PMC13557033 42719339

[70] Zhu J, Patel R, Trofka A, Harfe BD, Mackem S. Sonic hedgehog is not a limb morphogen but acts as a trigger to specify all digits in mice. Dev Cell. 2022;57(17):2048-2062.e4. doi: 10.1016/j.devcel.2022.08.008 PMC9709693 35977544

[71] Busby L, Aceituno C, McQueen C, Rich CA, Ros MA, Towers M. Sonic hedgehog specifies flight feather positional information in avian wings. Development. 2020;147(9):dev188821. doi: 10.1242/dev.188821 32376617 PMC7225127

[72] Neumann CJ, Grandel H, Gaffield W, Schulte-Merker S, Nüsslein-Volhard C. Transient establishment of anteroposterior polarity in the zebrafish pectoral fin bud in the absence of sonic hedgehog activity. Development. 1999;126(21):4817–26. doi: 10.1242/dev.126.21.4817 10518498

[73] Nachtrab G, Kikuchi K, Tornini VA, Poss KD. Transcriptional components of anteroposterior positional information during zebrafish fin regeneration. Development. 2013;140(18):3754–64. doi: 10.1242/dev.098798 23924636 PMC3754474

[74] Autumn M, Hu Y, Zeng J, McMenamin SK. Growth patterns of caudal fin rays are informed by both external signals from the regenerating organ and remembered identity autonomous to the local tissue. Dev Biol. 2024;515:121–8. 10.1016/j.ydbio.2024.07.008 39029570 PMC11361315

[75] Stewart S, Yette GA, Le Bleu HK, Henner AL, Braunstein JA, Chehab JW, Harms MJ, et al. Skeletal geometry and niche transitions restore organ size and shape during zebrafish fin regeneration . bioRxiv. 2019:606970. 10.1101/606970

[76] Adachi U, Koita R, Seto A, Maeno A, Ishizu A, Oikawa S, et al. Teleost Hox code defines regional identities competent for the formation of dorsal and anal fins. Proc Natl Acad Sci U S A. 2024;121(25):e2403809121. doi: 10.1073/pnas.2403809121 38861596 PMC11194558

[77] Lewandowski JP, Du F, Zhang S, Powell MB, Falkenstein KN, Ji H. Spatiotemporal regulation of GLI target genes in the mammalian limb bud. Dev Biol. 2015;406(1):92–103. doi: 10.1016/j.ydbio.2015.08.001 26238476 PMC4587286

[78] Litingtung Y, Dahn RD, Li Y, Fallon JF, Chiang C. Shh and Gli3 are dispensable for limb skeleton formation but regulate digit number and identity. Nature. 2002;418(6901):979–83. doi: 10.1038/nature01033 12198547

[79] Nakamura T, Gehrke AR, Lemberg J, Szymaszek J, Shubin NH. Digits and fin rays share common developmental histories. Nature. 2016;537(7619):225–8. doi: 10.1038/nature19322 27533041 PMC5161576

[80] Freitas R, Zhang G, Cohn MJ. Evidence that mechanisms of fin development evolved in the midline of early vertebrates. Nature. 2006;442(7106):1033–7. doi: 10.1038/nature04984 16878142

[81] Lettice LA, Devenney P, De Angelis C, Hill RE. The conserved sonic Hedgehog limb enhancer consists of discrete functional elements that regulate precise spatial expression. Cell Rep. 2017;20(6):1396–408. doi: 10.1016/j.celrep.2017.07.037 28793263 PMC5561167

[82] Sakamoto K, Onimaru K, Munakata K, Suda N, Tamura M, Ochi H, et al. Heterochronic shift in Hox-mediated activation of sonic hedgehog leads to morphological changes during fin development. PLoS One. 2009;4(4):e5121. doi: 10.1371/journal.pone.0005121 19365553 PMC2664896

[83] Schartl M, Kneitz S, Ormanns J, Schmidt C, Anderson JL, Amores A, et al. The developmental and genetic architecture of the sexually selected male ornament of swordtails. Curr Biol. 2021;31(5):911-922.e4. doi: 10.1016/j.cub.2020.11.028 33275891 PMC8580132

[84] Boehm B, Westerberg H, Lesnicar-Pucko G, Raja S, Rautschka M, Cotterell J, et al. The role of spatially controlled cell proliferation in limb bud morphogenesis. PLoS Biol. 2010;8(7):e1000420. doi: 10.1371/journal.pbio.1000420 20644711 PMC2903592

[85] Towers M, Mahood R, Yin Y, Tickle C. Integration of growth and specification in chick wing digit-patterning. Nature. 2008;452(7189):882–6. doi: 10.1038/nature06718 18354396

[86] Zhu J, Nakamura E, Nguyen M-T, Bao X, Akiyama H, Mackem S. Uncoupling Sonic hedgehog control of pattern and expansion of the developing limb bud. Dev Cell. 2008;14(4):624–32. doi: 10.1016/j.devcel.2008.01.008 18410737 PMC8284562

[87] Lopez-Rios J, Speziale D, Robay D, Scotti M, Osterwalder M, Nusspaumer G. GLI3 constrains digit number by controlling both progenitor proliferation and BMP-dependent exit to chondrogenesis. Dev Cell. 2012;22(4):837–48. doi: 10.1016/j.devcel.2012.03.002 22465667 PMC4486391

[88] Bailon-Zambrano R, Keating MK, Sales EC, Nichols AR, Gustafson GE, Hopkins CA. The sclerotome is the source of the dorsal and anal fin skeleton and its expansion is required for median fin development. Development. 2024. doi: dev.203025 10.1242/dev.203025 PMC11664171 39575996

[89] Ma RC, Kocha KM, Méndez-Olivos EE, Ruel TD, Huang P. Origin and diversification of fibroblasts from the sclerotome in zebrafish. Dev Biol. 2023;498:35–48. 10.1016/j.ydbio.2023.03.004 36933633

[90] Ma RC, Jacobs CT, Sharma P, Kocha KM, Huang P. Stereotypic generation of axial tenocytes from bipartite sclerotome domains in zebrafish. PLoS Genet. 2018;14(11):e1007775. doi: 10.1371/journal.pgen.1007775 30388110 PMC6235400

[91] Thieme P, Warth P, Moritz T. Development of the caudal-fin skeleton reveals multiple convergent fusions within Atherinomorpha. Front Zool. 2021;18(1):20. doi: 10.1186/s12983-021-00408-x 33902629 PMC8077867

[92] Hoshino K. Homologies of the caudal fin rays of Pleuronectiformes (Teleostei). Ichthyol Res. 2001;48(3):231–46. 10.1007/s10228-001-8141-6

[93] Waldmann L, Leyhr J, Zhang H, Allalou A, Öhman-Mägi C, Haitina T. The role of Gdf5 in the development of the zebrafish fin endoskeleton. Dev Dyn. 2022;251(9):1535–49. doi: 10.1002/dvdy.399 34242444

[94] Lauder GV. Function of the caudal fin during locomotion in fishes: kinematics, flow visualization, and evolutionary patterns. Am Zool. 2000;40(1):101–22.

[95] Davesne D, Friedman M, Schmitt AD, Fernandez V, Carnevale G, Ahlberg PE, et al. Fossilized cell structures identify an ancient origin for the teleost whole-genome duplication. Proc Natl Acad Sci. 2021;118(30):e2101780118. doi: 10.1073/pnas.2101780118 PMC8325350 34301898

[96] Song J, Zhong Y, Du R, Yin L, Ding Y. Tail shapes lead to different propulsive mechanisms in the body/caudal fin undulation of fish. Proc Inst Mech Eng C: J Mech Eng Sci. 2021;235(2):351–64.

[97] Fricke R, Durville P. Coris flava, a new deep water species of wrasse from La Réunion, southwestern Indian Ocean (Teleostei: Labridae). Fishtaxa-J Fish Taxon. 2021;22.

[98] Randall JE, Kuiter RH. Three new labrid fishes of the genus Coris from the Western Pacific. Zool Scr. 1982.

[99] Salis P, Roux N, Huang D, Marcionetti A, Mouginot P, Reynaud M. Thyroid hormones regulate the formation and environmental plasticity of white bars in clownfishes. Proc Natl Acad Sci U S A. 2021;118(23):e2101634118. doi: 10.1073/pnas.2101634118 PMC8201804 34031155

[100] DeLaurier A, Eames BF, Blanco-Sánchez B, Peng G, He X, Swartz ME, et al. Zebrafish sp7:EGFP: a transgenic for studying otic vesicle formation, skeletogenesis, and bone regeneration. Genesis. 2010;48(8):505–11. doi: 10.1002/dvg.20639 20506187 PMC2926247

[101] Kirby BB, Takada N, Latimer AJ, Shin J, Carney TJ, Kelsh RN, et al. In vivo time-lapse imaging shows dynamic oligodendrocyte progenitor behavior during zebrafish development. Nat Neurosci. 2006;9(12):1506–11. doi: 10.1038/nn1803 17099706

[102] Bouldin CM, Snelson CD, Farr GH, Kimelman D. Restricted expression of cdc25a in the tailbud is essential for formation of the zebrafish posterior body. Genes Dev. 2014;28(4):384–95. 24478331 10.1101/gad.233577.113 PMC3937516

[103] van Eeden FJ, Granato M, Schach U, Brand M, Furutani-Seiki M, Haffter P, et al. Genetic analysis of fin formation in the zebrafish, *Danio rerio*. Development. 1996;123:255–62. doi: 10.1242/dev.123.1.255 9007245

[104] Walker M, Kimmel C. A two-color acid-free cartilage and bone stain for zebrafish larvae. Biotech Histochem. 2007;82(1). 10.1080/10520290701333558 17510811

[105] Schindelin J, Arganda-Carreras I, Frise E, Kaynig V, Longair M, Pietzsch T, et al. Fiji: an open-source platform for biological-image analysis. Nat Methods. 2012;9(7):676–82. doi: 10.1038/nmeth.2019 22743772 PMC3855844

[106] Sehring I, Mohammadi HF, Haffner-Luntzer M, Ignatius A, Huber-Lang M, Weidinger G. Huber-Lang M, Weidinger G. Zebrafish fin regeneration involves generic and regeneration-specific osteoblast injury responses. eLife. 2022;11:e77614. doi: 10.7554/eLife.77614 35748539 PMC9259016

