# Supplementary figures and images for: Caudal fin shape imprinted during late zebrafish embryogenesis is re-patterned by the Sonic hedgehog pathway
Source: PLoS Biol. 2025 Aug 25;23(8):e3003336. doi: 10.1371/journal.pbio.3003336 (PMC12396763; doi:10.1371/journal.pbio.3003336)

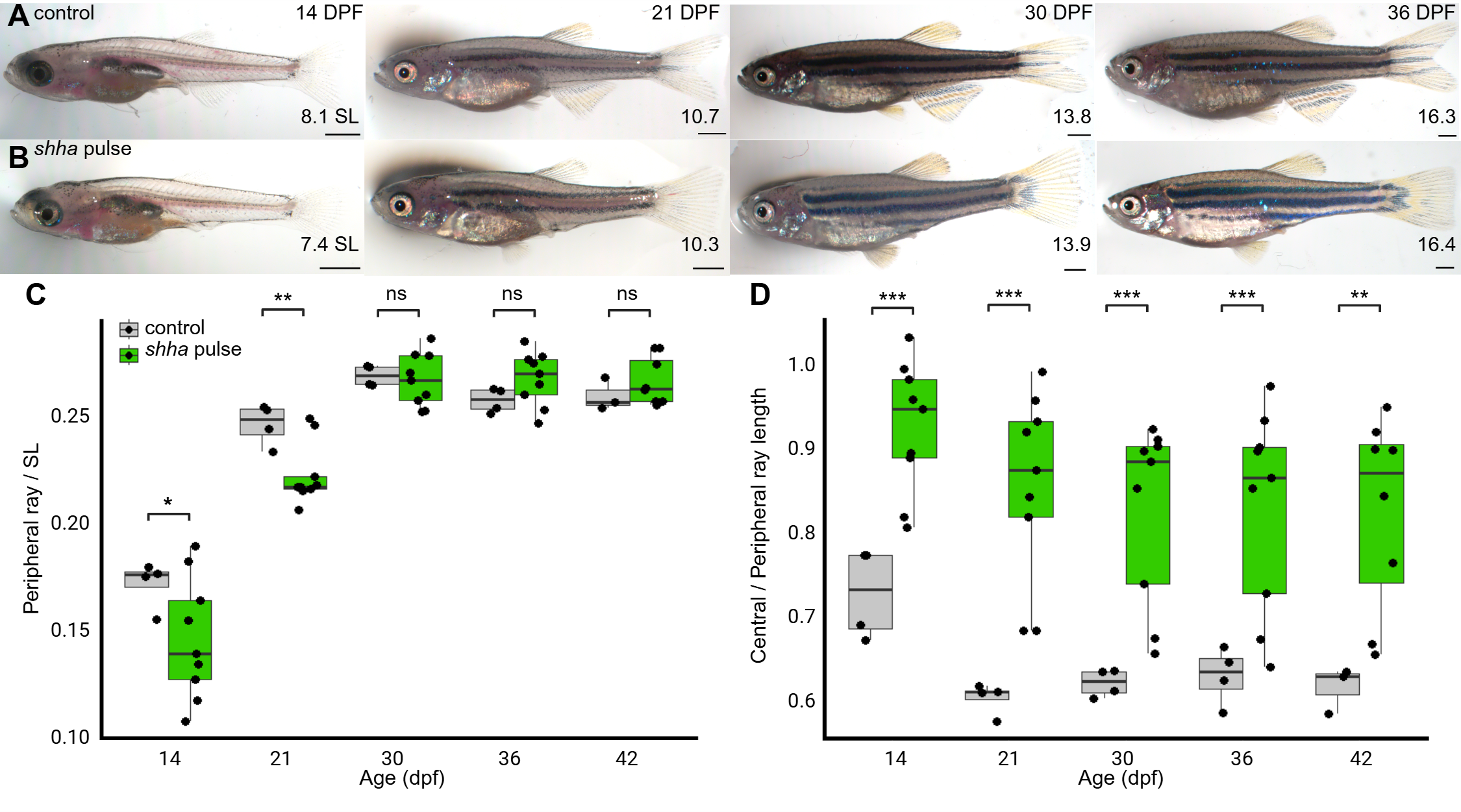

Supplement: S1 Fig — (A–B) Whole body images of (A) control and (B) transgenic clutch mates treated with shha pulse. Scale bars, 500 µm. (C) The overall length of the caudal fin (as measured by the length of the peripheral 2nd dorsal ray) relative to the standard length (SL), tracked in individuals from the same clutch for the first 6 weeks of development. By 30 dpf, truncate fins were the same length as the forked fins of controls. (D) The difference in caudal fin shape between conditions is evident by 14 dpf. Significance within each time point determined by Welch’s two-tailed T-tests. The data underlying the graphs shown in the figure can be found in S1 Data and the summary statistics in S2 Data. (TIF) [file pbio.3003336.s001.tif]

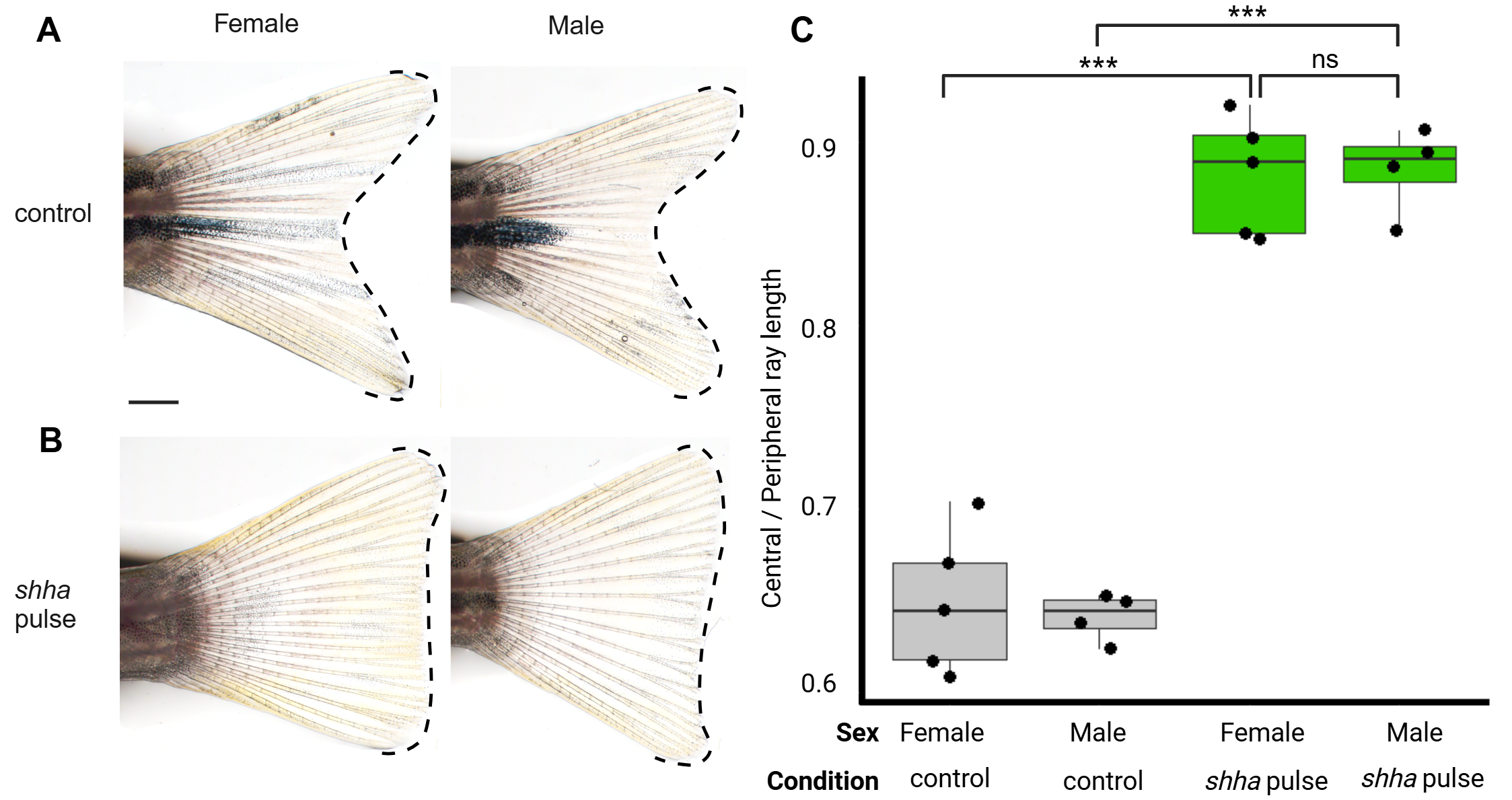

Supplement: S2 Fig — Representative caudal fins of male and female (A) control and (B) shh pulse-treated individuals. Scale bar, 1 mm. (C) There was no difference in fin shape between sexes in either control or shh pulse-treated fish. Significance determined by ANOVA followed by Tukey’s post hoc test. The data underlying the graphs shown in the figure can be found in S1 Data and the summary statistics in S2 Data. (TIF) [file pbio.3003336.s002.tif]

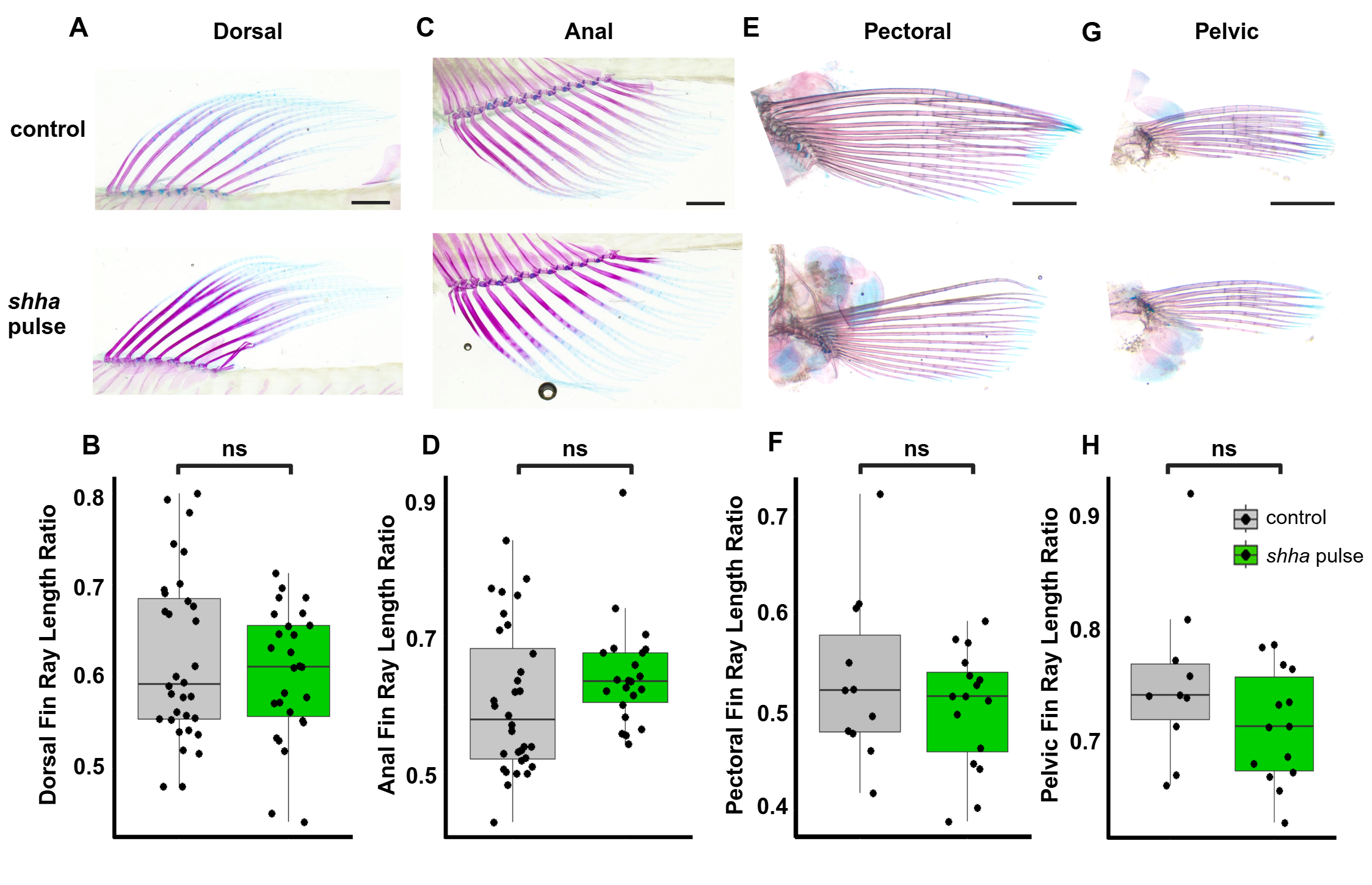

Supplement: S3 Fig — Shapes of fins were quantified as the ratio in lengths of the shortest bifurcating ray to the longest bifurcating ray. Adult dorsal (A–B), anal (C–D), pectoral (E–F) and pelvic fins (G–H) showed no differences in shape after shha pulse compared to control clutch mates. Scale bars, 1 mm. Significance determined using Welch’s two-tailed T-tests. The data underlying the graphs shown in the figure can be found in S1 Data and the summary statistics in S2 Data. (TIF) [file pbio.3003336.s003.tif]

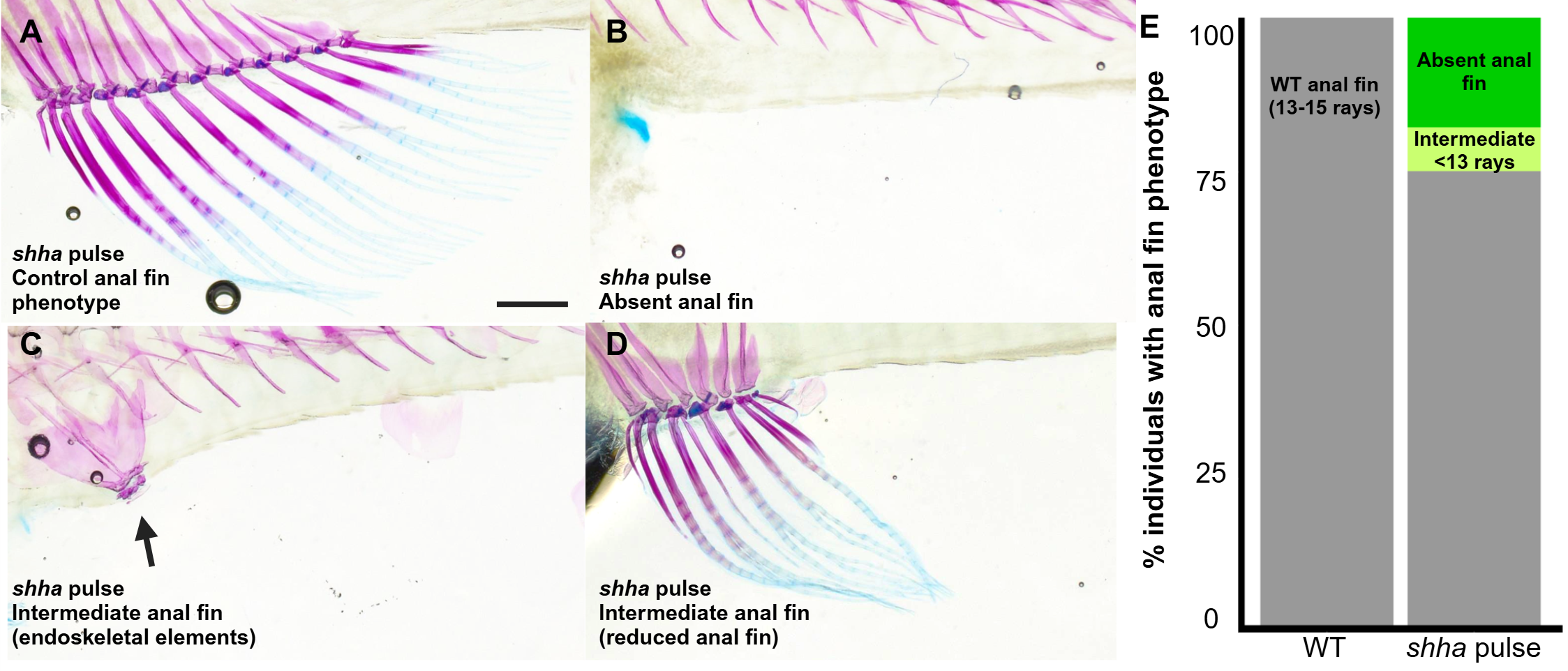

Supplement: S4 Fig — (A) In approximately 75% of individuals treated with shha pulse, the anal fin develops with a shape and size comparable to that those in control individuals. (B) In approximately 20% of fish treated with shha pulse, no anal fin develops. (C–D) In approximately 5% of individuals treated with shha pulse, a reduced number of endoskeletal bones (black arrow) and fin rays develop. Scale bar, 1 mm. Phenotype percentages displayed in (E). (TIF) [file pbio.3003336.s004.tif]

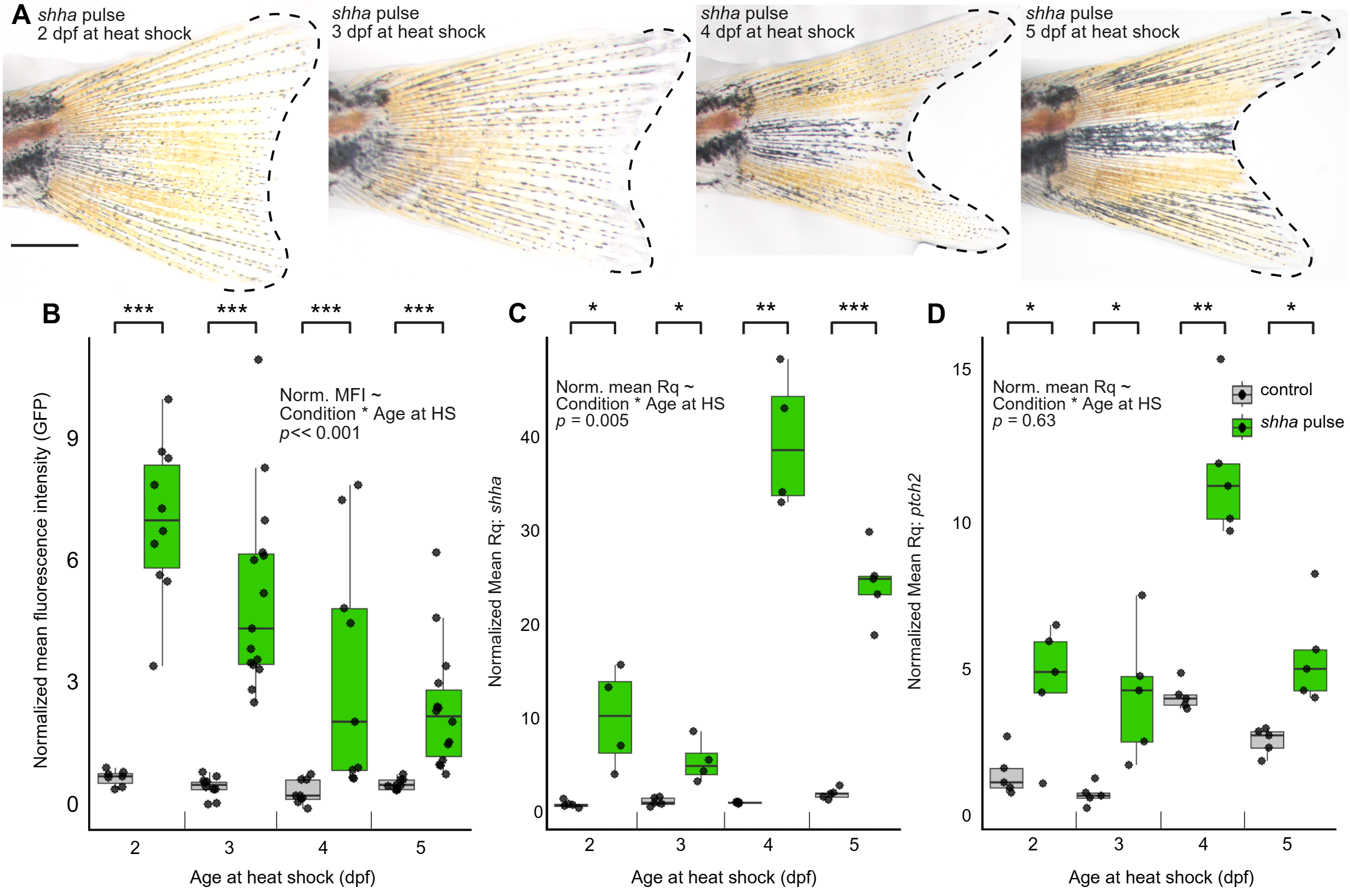

Supplement: S5 Fig — (A) Caudal fins of fish treated with shha pulse at different days post-fertilization. Dashed outlines indicate the overall shape of the fins. Scale bar, 500 µm. (B) Individuals were heat shocked at different days post-fertilization, and mean fluorescence intensity was measured 16–18 hrs later. Each data point represents an individual larva. GFP was visible in transgenic larvae after each heat shock treatment, regardless of day of heat shock. (C–D) Individuals were heat-shocked at different days post-fertilization and expression of shha (C) and ptch2 (D) were measured by qRT-PCR. Each datapoint represents a biological replicate of 3 pooled larvae, collected at approximately 6 hrs after heat shock, normalized to a single replicate in the control group. Significance within each time point determined by Welch’s two-tailed T-tests, and the correlation between the conditional readout and age at heat shock determined by linear-mixed effects model. The data underlying the graphs shown in the figure can be found in S1 Data and the summary statistics in S2 Data. (TIF) [file pbio.3003336.s005.tif]

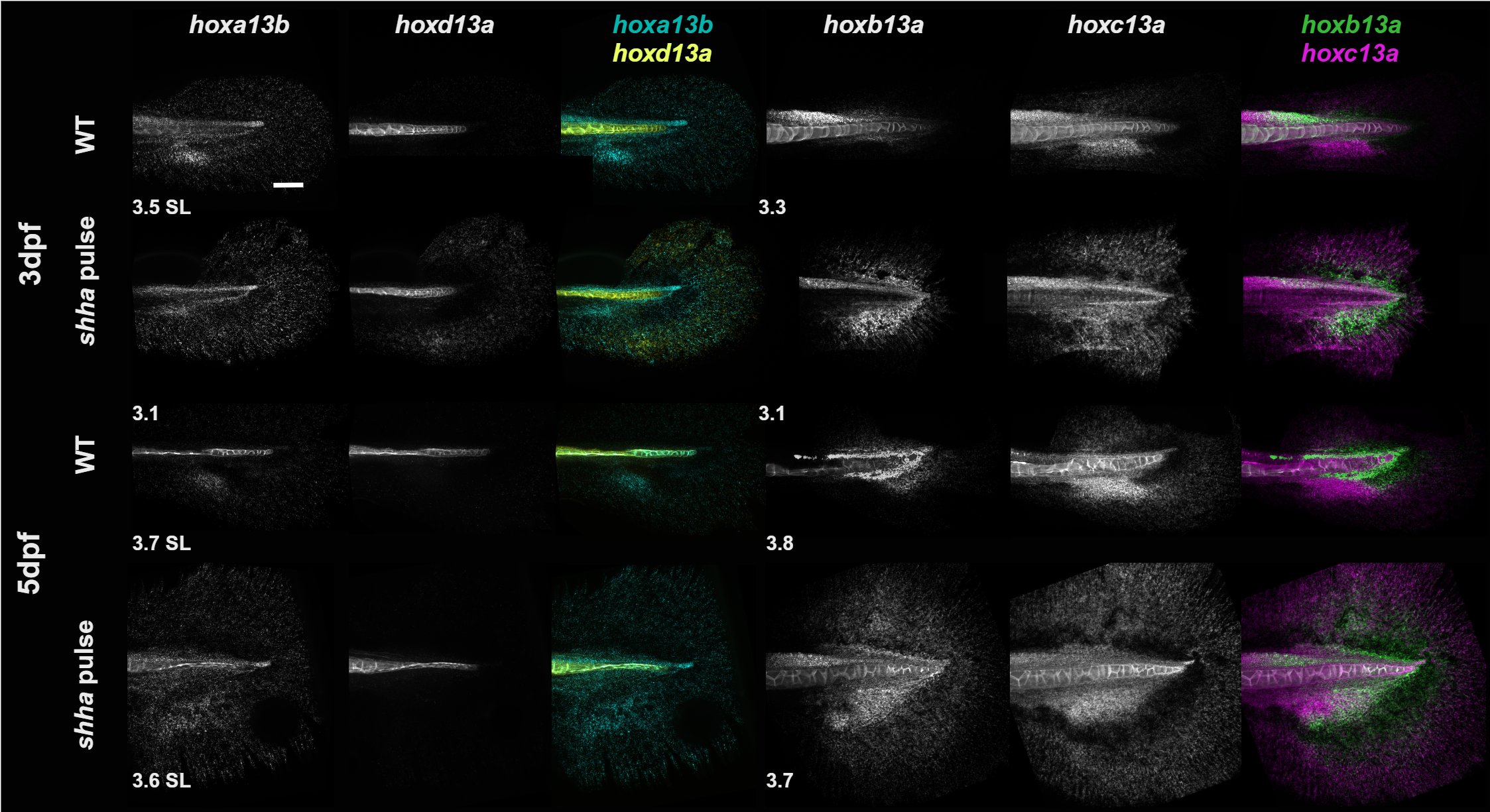

Supplement: S6 Fig — Caudal fins of WT controls and individuals treated with shha pulse caudal fin folds at 3 and 5 days post-fertilization. Merged images of hoxa13b + hoxd13a and hoxb13a + hoxc13a are displayed next to the single-channel images. A minimum of 3 individuals were examined for each condition and time point. Small white arrows indicate the posterior end of the caudal artery. Standard lengths reported are corrected after fixation [19]. Scale bar, 100 µm. (TIF) [file pbio.3003336.s006.tif]

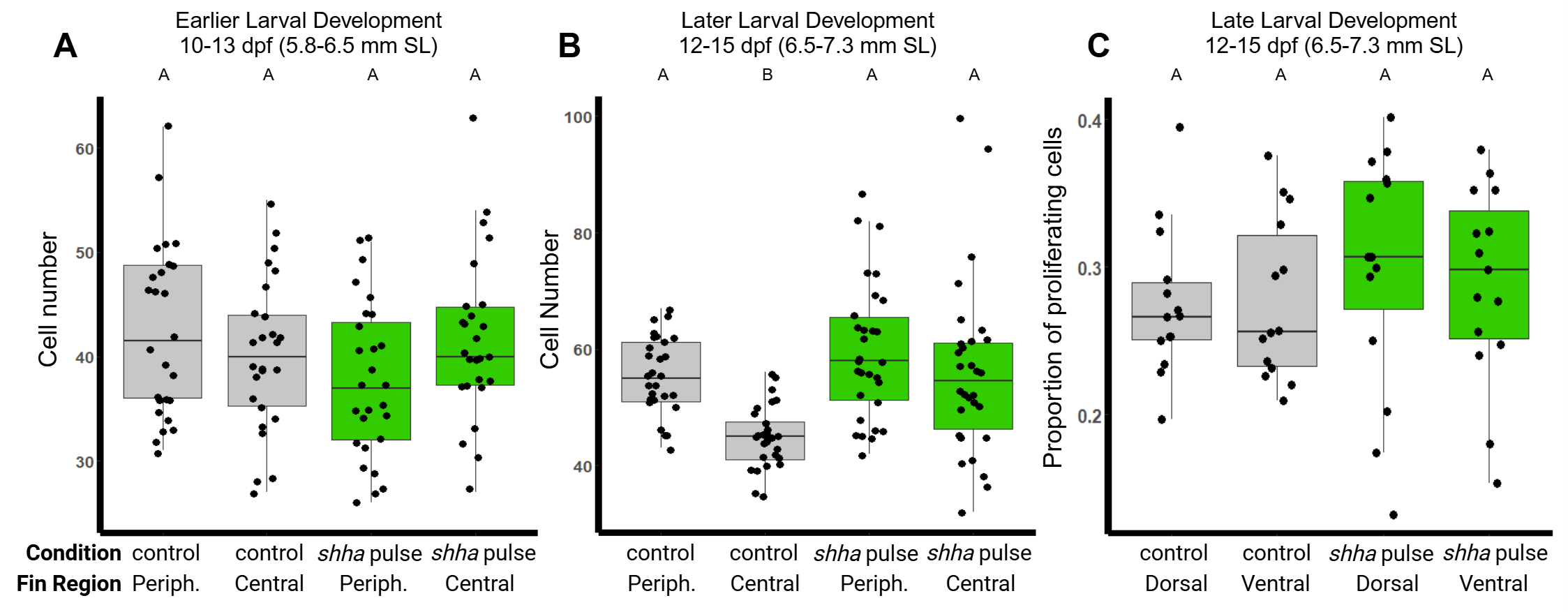

Supplement: S7 Fig — (A) At earlier stages of larval development (5.8–6.5 SL) there is no difference in cell number between central and peripheral fin regions in either condition (non-transgenic control or shha pulse). (B) At later stages of larval development (6.5–7.2 SL), control larvae show relatively fewer cells in central regions, but larvae treated with shha pulse show similar cell numbers in central and peripheral regions. (C) Dorsal and ventral regions of developing larval fins do not show different proportions of proliferating cells. Significance determined by linear mixed-effects model followed by Tukey’s post hoc test; statistically indistinguishable groups are shown with the same letter (threshold for significance p < 0.05). The data underlying the graphs shown in the figure can be found in S1 Data and the summary statistics in S2 Data. (TIF) [file pbio.3003336.s007.tif]

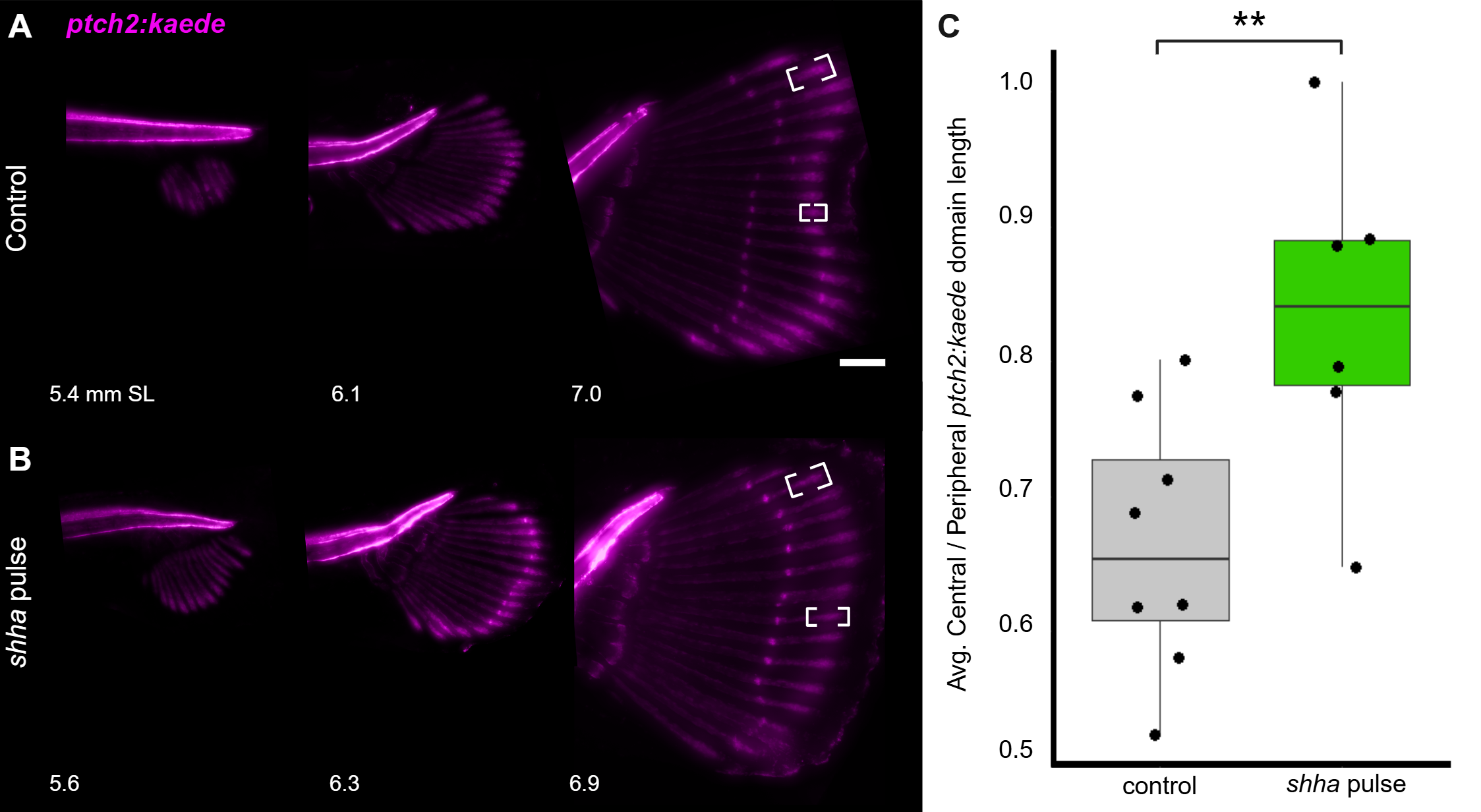

Supplement: S8 Fig — (A–B) Repeated tracking of individual Tg(ptch2:kaede) larvae during early caudal fin development. Image series performed on a minimum of 7 larvae per condition. Distal domains of expression in peripheral and central rays utilized for quantification in (C) shown in brackets. Scale bar, 100 µm. (C) The domain length ratio of the central to the peripheral region between conditions. Significance determined using Welch’s two-tailed t test. The data underlying the graphs shown in the figure can be found in S1 Data and the summary statistics in S2 Data. (TIF) [file pbio.3003336.s008.tif]
