# Supplementary material for: Caudal fin shape imprinted during late zebrafish embryogenesis is re-patterned by the Sonic hedgehog pathway
Source: PLoS Biol. 2025 Aug 25;23(8):e3003336. doi: 10.1371/journal.pbio.3003336 (PMC12396763; doi:10.1371/journal.pbio.3003336)
Supplement: S1 Table — (DOCX) [file pbio.3003336.s009.docx]

**S1 Table: Primer sets used for qPCR and RT-qPCR**

| Ptch2_F cDNA | TGTGCTGTTTCTACAGTCCCTG |
| --- | --- |
| Ptch2_R cDNA | GCACGCTGATGGTTGTCATT |
| Shha_F cDNA | AGAGCCGGACAAAAGGTGAT |
| Shha_R cDNA | AATGGTCCCATGTGCAGTCA |
| ActB1_F cDNA | CGACCAGAAGCGTACAGAGA |
| ActB1_R cDNA | AATCCCAAAGCCAACAGAGA |
| EGFP_F gDNA | ACGACGGCAACTACAAGACC |
| EGFP_R gDNA | TTGCCGTCCTCCTTGAAGTC |
| Actb1_F gDNA | GATGCGGAAACTGGAAAGGG |
| Actb1_R gDNA | GGAGGGCAAAGTGGTAAACG |
